# Intussusceptive Vascular Remodeling Precedes Pathological Neovascularization

**DOI:** 10.1161/ATVBAHA.118.312190

**Published:** 2019-05-09

**Authors:** Zaheer Ali, Anthony Mukwaya, Antje Biesemeier, Maria Ntzouni, Daniel Ramsköld, Sarantis Giatrellis, Parviz Mammadzada, Renhai Cao, Anton Lennikov, Michele Marass, Claudia Gerri, Camilla Hildesjö, Michael Taylor, Qiaolin Deng, Beatrice Peebo, Luis del Peso, Anders Kvanta, Rickard Sandberg, Ulrich Schraermeyer, Helder Andre, John F. Steffensen, Neil Lagali, Yihai Cao, Julianna Kele, Lasse Dahl Jensen

**Affiliations:** 1 From the Division of Cardiovascular Medicine, Department of Medical and Health Sciences (Z.A., L.D.J.), Linkoping University, Sweden; 2 Division of Ophthalmology, Department of Clinical and Experimental Medicine (A.M., A.L., B.P., N.L.), Linkoping University, Sweden; 3 Electronmicroscopy and Histology Laboratory, Faculty of Medicine (M.N.), Linkoping University, Sweden; 4 Division of Surgery, Orthopedics and Oncology, Department for Clinical and Experimental Medicine (C.H.), Linkoping University, Sweden; 5 Experimental Vitreoretinal Surgery, Center for Ophthalmology, University of Tuebingen, Germany (A.B., U.S.); 6 Department of Cell and Molecular Biology (D.R., S.G., R.S.), Karolinska Institutet, Stockholm, Sweden; 7 Department of Clinical Neuroscience, Section for Ophthalmology and Vision, St. Erik Eye Hospital (P.M., A.K., H.A.), Karolinska Institutet, Stockholm, Sweden; 8 Department of Microbiology, Tumor and Cell Biology (R.C., Y.C.), Karolinska Institutet, Stockholm, Sweden; 9 Department of Physiology and Pharmacology (Q.D., J.K.), Karolinska Institutet, Stockholm, Sweden; 10 Department of Developmental Genetics, Max Planck Institute for Lung and Heart Research, Bad Nauheim, Germany (M.M., C.G.); 11 Pharmaceutical Sciences Division, School of Pharmacy, University of Wisconsin-Madison (M.T.); 12 Department of Biochemistry, Universidad Autónoma de Madrid, Spain (L.d.P.); 13 Instituto de Investigaciones Biomédicas Alberto Sols, CSIC-UAM Madrid, Spain (L.d.P.); 14 Marine Biological Section, Biological Institute, University of Copenhagen, Helsingor, Denmark (J.F.S.).

**Keywords:** choroidal neovascularization, hypoxia, intussusception, macular degeneration, zebrafish

## Abstract

**Objective—:**

Pathological neovascularization is crucial for progression and morbidity of serious diseases such as cancer, diabetic retinopathy, and age-related macular degeneration. While mechanisms of ongoing pathological neovascularization have been extensively studied, the initiating pathological vascular remodeling (PVR) events, which precede neovascularization remains poorly understood. Here, we identify novel molecular and cellular mechanisms of preneovascular PVR, by using the adult choriocapillaris as a model.

**Approach and Results—:**

Using hypoxia or forced overexpression of VEGF (vascular endothelial growth factor) in the subretinal space to induce PVR in zebrafish and rats respectively, and by analyzing choriocapillaris membranes adjacent to choroidal neovascular lesions from age-related macular degeneration patients, we show that the choriocapillaris undergo robust induction of vascular intussusception and permeability at preneovascular stages of PVR. This PVR response included endothelial cell proliferation, formation of endothelial luminal processes, extensive vesiculation and thickening of the endothelium, degradation of collagen fibers, and splitting of existing extravascular columns. RNA-sequencing established a role for endothelial tight junction disruption, cytoskeletal remodeling, vesicle- and cilium biogenesis in this process. Mechanistically, using genetic gain- and loss-of-function zebrafish models and analysis of primary human choriocapillaris endothelial cells, we determined that HIF (hypoxia-induced factor)-1α-VEGF-A-VEGFR2 signaling was important for hypoxia-induced PVR.

**Conclusions—:**

Our findings reveal that PVR involving intussusception and splitting of extravascular columns, endothelial proliferation, vesiculation, fenestration, and thickening is induced before neovascularization, suggesting that identifying and targeting these processes may prevent development of advanced neovascular disease in the future.

## Abstract 2

Supplemental Digital Content is available in the text.

**Highlights**

- Early pathological vascular remodeling (PVR) and leakage of the choriocapillaris is a vascular priming mechanism associated with age-related macular degeneration that can be modeled by hypoxia treatment in adult zebrafish and closely resembles PVR phenotypes in rodents and age-related macular degeneration patients.
- PVR involves endothelial cell proliferation, cytoskeletal reorganization, tight junction remodeling, fenestration, vesicle and cilium biogenesis, loosening of smooth muscle cell contracts, and degradation of collagen fibers.
- These processes culminate in the production of immature intussusceptive pillars (nonproductive intussusception) as well as both generation of new, mature intussusceptive pillars, and splitting of existing extravascular columns (productive intussusception and vessel fusion), which is central to the PVR response also in rodents and wet age-related macular degeneration patients.
- PVR is dependent of signaling through the HIF (hypoxia-induced factor)-1-VEGF (vascular endothelial growth factor)-VEGFR2 axis and may be targeted using available anti-VEGF approaches.

Common diseases including cancer, cardiovascular disorders, chronic inflammatory diseases, and retinopathies are associated with pathological and ectopic vessel growth (neovascularization), which in most cases implies a switch to more advanced disease stages associated with poor prognosis.^1^ For example, the growth of primary or metastatic tumor nodules to clinically detectable masses,^2^ growth and destabilization of atherosclerotic plaques before rupture,^3^ synovitis and joint hypertrophy in rheumatoid arthritis,^4^ macular edema, retinal detachment and vision loss in diabetic retinopathy (DR),^5^ and age-related macular degeneration (AMD)^6^ all depend on pathological neovascularization. Ongoing neovascularization is associated with pathological vascular phenotypes including lack of arterial-venous differentiation, pericyte coverage, and endothelial tightness resulting in poor perfusion, high leakage and interstitial fluid pressure, and reduced drug delivery and efficacy in the affected tissue.^7,8^ As such, targeting pathological neovascularization has great potential for treatment of many common, serious diseases and is likely most effective if deployed early and preferably as a preventive strategy. While intensive research over the past several decades has led to tremendous progress in our understanding of the mechanisms and processes involved in ongoing pathological neovascularization,^2^ the molecular mechanisms involved in the initiating events, that is, early pathological vascular remodeling (PVR), remains underinvestigated and poorly understood. As such, there are currently no specific targets known, or treatments available, for preventing the switch from a pathologically activated vasculature to neovascularization. Furthermore, the cellular and physiological changes involved during preneovascular activation are not known, preventing early diagnosis of patients at high risk of progression to neovascular disease stages.

**See cover image**

The choriocapillaris is the vasculature located posterior to and closely associated with the retinal pigment epithelium (RPE) and photoreceptors of the outer retina. Choriocapillaris growth through the protective RPE-shield known as Bruch’s membrane, a process referred to as choroidal neovascularization (CNV), causes the progression of AMD to advanced, so-called exudative or wet, stages.^9^ As AMD is one of the most prevalent neovascularization-associated diseases and the most common cause of blindness worldwide, massive efforts have been dedicated to developing antineovascular treatments for this disorder. As a result, antineovascular drugs including bevacizumab, ranibizumab, and aflibercept, have been developed, which are effectively restoring vision in the short term for many wet AMD patients.^10^ However, once choriocapillaris have penetrated Bruch’s membrane and grown into the retina, which is the clinical indication for starting antineovascular drug treatment, it is no longer possible to achieve full and persistent regression of pathological blood vessels. As the ectopic, subretinal vasculature that remains in patients after antineovascular treatment are thought to be involved in recurrence, patients that have already developed wet AMD are at risk of progressing after a period of therapeutic benefit.^9^ Similar problems are observed in other neovascularization-associated diseases including DR, other chronic inflammatory disorders, and various types of cancer currently treated with antineovascular drugs.^11,12^ As such, identifying early, actionable targets for pathological transformation of the involved vasculatures preceding neovascularization would potentially enable preventive rather than therapeutic measures to reduce the risk of disease progression and morbidity.

Intussusception is a form of vascular remodeling and growth involving the splitting of vessels by extending cellular processes generated by endothelial and vascular mural cells, into the vascular lumen.^13^ As these processes anastomose, they form transluminal, intussusceptive pillars (ISPs) that then expand by growing in circumference, fusing with other ISPs and remodel into extravascular columns of interstitial tissue. Eventually, this leads to the original vessel having transformed into vascular loops^14^ or by longitudinal intussusception into 2 separate vessels.^13^ The developing choriocapillaris, lung, and kidney vasculatures, as well as pathological growth of vessels in tumors and the cornea, are thought to use intussusception as a mechanism of vascular growth and remodeling.^14–17^ In these cases, intussusception is considered an alternative to sprouting angiogenesis^17^ or to mediate vascular maturation and remodeling following a period of ongoing angiogenesis.^14^ Postneovascular intussusception might be used as a mechanism of evading antineovascular therapies, as vessels undergoing intussusception are stabilized and better able to resist for example anti-VEGF (vascular endothelial growth factor) drugs.^14,18,19^ As such it has been suggested that in the context of high levels of VEGF-signaling, vessels use sprouting angiogenesis as their mechanism of growth, whereas in the context of reduced or inhibited VEGF-signaling they switch to intussusception.^17,19,20^ Intussusception may, however, also be induced by VEGF-signaling,^21^ implying that the role of VEGF in regulating vessel sprouting versus intussusception is still incompletely understood and may be context-dependent.

Hypoxia is a main pathological driver of neovascularization in disease.^22,23^ In hypoxic tissues, the catalytic activity of the oxygen sensors prolyl hydroxylase domain (PHD)-1–3 and factor inhibiting HIF (hypoxia-induced factor; FIH) are reduced leading to impaired hydroxylation of HIFs resulting in their stabilization and unabated transcriptional activity.^24^ Several vascular growth factors including VEGF are under control of HIFs and therefore increased in response to hypoxia.^22^ VEGF, in turn, activate signaling through type 1 tyrosine kinase receptors, primarily VEGF receptor-2 expressed by endothelial cells (ECs), leading to vascular leakage and growth.^22,25^ While HIF-1α is critical for acute responses to hypoxia, HIF-2α is important for responses to prolonged hypoxia^26,27^ and the transcriptomic changes induced by hypoxia and HIFs are highly context-dependent.^28,29^ While a number of sophisticated animal models have attempted to address the complex issue of hypoxia-induced pathological changes to blood vessels indirectly by for example increasing the oxygen consumption in the RPE or reducing endothelial HIF-1α–signaling,^30–32^ it has not been possible to directly induce and control tissue hypoxia per se, in rodents.

Zebrafish are highly amenable to hypoxia studies as they are naturally hypoxia-tolerant and survive in severely hypoxic conditions for prolonged periods of time.^22,33–35^ In addition, zebrafish respond to complex pathophysiological stimuli, including hypoxia, in a manner that strongly resembles human disease. For example, human tumor cells implanted in zebrafish induce angiogenesis, disseminate and exhibit drug responses identical to those of the patient that donated the cells,^36–38^ and these processes are regulated by tumor hypoxia.^22,39,40^ In eye research, zebrafish models are particularly interesting as diabetic zebrafish exhibit cone-degeneration and vascular changes in the retinal vasculature similar to early stages of DR^41^ and hypoxia treatment induces retinal neovascularization by sprouting of new retinal capillaries similar to what is seen in patients with late-stage, proliferative DR.^34^ Similarly, hypoxia treatment of adult zebrafish has been implicated in opening up lymphatic vessels for perfusion with blood as a mechanism of rapidly increasing blood delivery to the tissues.^42^ While the retinal vasculature and large choroidal vessels have been studied in zebrafish,^43–45^ the choriocapillaris has, however, not been identified.

In this study, we identify and thoroughly characterize the adult zebrafish choriocapillaris and use this vasculature as a model for identifying a hypoxia-induced PVR mechanism that precedes CNV. Using transmission electron microscopy (TEM), confocal microscopy, histological analysis, and EC transcriptomics, we show that hypoxia treatment leads to robust endothelial proliferation, (productive) intussusception and formation of immature endothelial luminal processes (ELPs) that do not anastomose to form ISPs (nonproductive intussusception), as well as splitting of existing extravascular columns. Remodeled choriocapillaris furthermore exhibited partially dissolved tight junctions, loosened mural cell contacts, increased fenestration, and increased permeability. Hypoxia-induced vascular leakage and productive and nonproductive intussusception required high levels of VEGF-signaling and the PVR phenotypes from the zebrafish model were recapitulated in a rat model of VEGF-induced choriocapillaris remodeling as well as in patients with advanced AMD. These findings establish intussusception as a novel mechanism involved in PVR preceding neovascularization in both animal models and patients.

## Materials and Methods

The data that support the findings of this study are available from the corresponding author on reasonable request.

### Zebrafish Strains and Their Maintenance

Tg(fli1a:EGFP)y1, Tg(kdrl:EGFP)s843, Tg(kdrl:DsRed2)pd27, Tg(acta2:EGFP)ca7, Tg(tagln:EGFP)p151, and Tg(gata1a:DsRed2)sd2 transgenic strains^46–51^ were obtained from ZIRC, Oregon. Tg(fli1ep:Gal4FF)ubs3,^52^ Tg(UAS:RFP), Tg(UAS:VE- -EGFP)ubs12,^53^ Tg(UAS:EGFP-ZO.1)ubs5,^54^ and Tg(UAS:EGFP-UCHD)ubs18^55^ transgenic strains were from the Affolter laboratory and the Tg(pdgfrb:mcitrine;kdrl:DsRed2),^56^ Hif1aa^−^^/−^;Hif1ab^−^^/−^^57^ and Hsp70:VEGFAA-DN^58^ zebrafish lines were from the Stainier laboratory. All strains and double or triple transgenic crosses between these stains were maintained at the Zebrafish facility at Linköping University, Linköping, Sweden following standard protocols.^39,59^ All the experimental procedures have been previously approved by the Linköping animal ethics committee.

### Hypoxia Exposure

The design of the hypoxia chamber and protocol for hypoxia exposure of adult zebrafish have been described previously.^33,34,40,42^ Briefly, adult zebrafish between 6 and 12 months of age were acclimatized to progressively decreasing oxygen concentrations in the water over a period of 2 to 3 days and then kept in water with a relative air saturation of 10% for 10 days.

Vegfaa-DN the fish were incubated for 1 hour at 37°C daily between day 4 and 10 of hypoxia exposure. DMH4 (Sigma Aldrich) was added to the water from a 1000X stock in DMSO to a final concentration of 1 µmol/L.

### Zebrafish Euthanasia and Dissection

Zebrafish were anesthetized with 0.04% MS-222 (Ethyl 3-aminobenzoate methane sulfonic acid salt 98%, Sigma Aldrich) for 5 minutes and transferred to ice cold water for an additional 5 minutes. Fish were fixed in 4% PFA (Sigma Aldrich) for 24 hours at 4°C, and the eyes were dissected to isolate the retina or choroid as described earlier^33,44^ under a dissection microscope (Nikon SMZ 1500).

### Evaluation of Vascular Leakiness

Vascular leakiness in adult zebrafish was evaluated essentially as described for embryos,^60^ by injection of 0.5 µL 70 kDa rhodamine-labeled, lysine-modified dextran (Invitrogen, United States) IP with a 10 µL syringe (Hamilton) under anesthesia with 0.02% MS-222. At 15 minutes after the injection, the zebrafish were euthanized and fixed in 4% PFA for 24 hours at 4°C. Eyes were then dissected as described above and visualized by confocal microscopy as described below.

### Fluorescence-Activated Cell Sorting

To obtain choroid ECs, adult fli1a:EGFP zebrafish were euthanized, and the choroids were isolated by manual dissection in ice-cold PBS (w/o Mg++ or Ca++, Gibco). Tissues were cut into fine pieces and placed in an equal volume of a mixture of collagenase (2.5 mg/mL Worthington Biochemical Corporation cat number LS004176) and DNase (30 U/μL, SIGMA, to a final of 0.04 mg/mL) in PBS (Mg++ and Ca++, Gibco) for 30 minutes on a shaker. Cells were filtered through a cell strainer (40 μm mesh, FALCON) followed by addition of 10% FBS:DMEM to a final volume of 6 mL and centrifugation at 800*g*, +4°C for 5 minutes. The supernatant was removed, and cells were recovered in 1.5 mL of cold PBS with 2% FBS before flow cytometry in a BD Influx (Becton Dickinson) cell sorter.

### RNA-Sequencing

ECs from control or hypoxia-treated zebrafish choroids were isolated by fluorescence-activated cell sorting (FACS). Cell samples (containing 30, 60, or all [>1000] EGFP-positive cells) were further processed for total RNA (RNeasy Micro Kit, Qiagen) followed by pre-RNA Sequencing procedures. Libraries were prepared using SMARTer Ultra Low Input RNA for Illumina Sequencing kit (Clontech) and sequenced on an Illumina HiSeq 2000. Reads were aligned with STAR (spliced transcripts alignment to a reference). As quality control, we calculated Spearman correlation between samples and removed the worst samples. All samples within each group were comparable after multiple testing correction. Raw data and expression values have been deposited to NCBI Sequence Read Archive with ID SRP056125 and Gene Expression Omnibus with ID GSE66848. False discovery rates were calculated by the Benjamini-Hochberg method.

Upregulated and downregulated genes from RNA-sequencing data were classified into functional annotation clusters using DAVID^61^ available at https://david.ncifcrf.gov/home.jsp. All the analysis parameters were kept at default. Only annotation clusters with enrichment score >1 were considered significant.

### Immunohistochemistry

Fixed eyes from adult zebrafish were washed 3× with PBS and further incubated in PBS for 2 hours at room temperature or 24 hours at 4°C. Retinal or choroid tissues were isolated by careful dissection and incubated in Proteinase K (20 µg/mL) for 5 minutes at room temperature on a rocking table. Tissues were then incubated for 30 minutes at room temperature in absolute methanol followed by 0.3% Triton–X PBS (PBS-Tx) for 30 minutes at room temperature on the rocking table followed by blocking in 3% milk in PBS-Tx overnight (16–24 hours) at 4°C. After washing 3× in PBS-Tx, tissues were incubated in primary antibodies Zo-1 (4 µg/mL, Invitrogen), VE-Cadherin (1:100 dilution),^62^ or monoclonal anti-GFAP (glial fibrillary acidic protein, 1:50 dilution, Sigma), diluted in 0.3% PBS-Tx 100 for 24 hours at 4°C. Tissues were then washed 3× in PBS-Tx 100, incubated in PBS-Tx for 90 minutes at +4°C, reblocked in 3% milk PBS-Tx 100 for 90 minutes at room temperature and incubated with secondary antibody; Alexa flour 555, goat anti-rabbit (1:200 dilution, Life Technologies) at a concentration of 10 µg/mL in blocking buffer, overnight at +4°C. Tissues were washed 3× in PBS-Tx and incubated overnight in blocking solution and finally mounted within viewing chambers in Vectashield (Vector).

For phalloidin staining, the samples were washed 3× for 5 minutes with 0.1% Tween-20 in PBS. The tissues were permeabilized in 2% PBS-Tx 100 for 1.5 hours at room temperature. Tissues were stained with phalloidin 5 U (1:40 dilution, Life Technologies) diluted in 2% PBS-Tx at 4°C overnight on a rocking table in the dark. The next day, tissue samples were washed 5× in 0.1% Tween-20 in the dark, followed by mounting as described above.

### Human Choroidal and Retinal EC Isolation, Hypoxia Treatment, and Quantitative PCR Analysis

Isolation of human choroidal and retinal ECs was described previously.^63^ Choroidal and retinal ECs were cultured and incubated either at normoxia (20% O_2_, 5% CO_2_) or exposed to 12 hours of hypoxia (1% O_2_, 5% CO_2_) in a humidified incubation chamber at 37°C when cells reached 80%–90% confluence. Total RNA was extracted from hypoxic and normoxic choroidal and retinal ECs (RNeasy minikit; QIAGEN). cDNA synthesis was performed from 500 ng total RNA (iScript cDNA Synthesis Kit BioRad Laboratories), and quantitative PCR was performed by iQ SYBR Green Supermix using MyiQ SingleColor Real-Time PCR Detection System (BioRad Laboratories). All protocols were performed according to the manufacturer’s instructions. Gene expression differences were calculated by the ΔΔCt method from 3 independent experiments, with hTBP (human TATA box-binding protein) as a reference gene. The primer sets were purchased from Eurogentec (BioNordika Sweden AB), and sequences are listed in the online-only Data Supplement.

### Electron Microscopy Sample Preparation

Specimens were fixed in a mixture of 2.5% glutaraldehyde and 4% formaldehyde in 0.1 mol/L Na cacodylate, pH 7.4, for 3 hours at room temperature. Specimens were washed with 0.1 mol/L Na cacodylate buffer and post fixed in 2% osmium tetroxide buffered in 0.1 mol/L Na cacodylate at 4°C. After 1-hour specimens were washed 2× with 0.1 mol/L Na cacodylate buffer and 3× with distilled water. Staining with 1% aqueous uranyl acetate was performed en block at 4°C for 1 hour. Specimens were then washed 3× with distilled water and dehydrated in a series of ascending concentration of ethanol and propylene oxide. Infiltration took place in 3 steps and finally the samples were embedded in Araldite 502/Embed 812 embedding medium at 60° for 24 hours (according to the manufacturer’s protocol, Electron Microscopy Science, Industry Road Hatfield, United States).

### Electron Microscopic Analysis

Blocks were trimmed and sectioned by using a Reichert Ultracut S, Leica ultramicrotome. Semithin sections (1 µm thickness) were stained with 1% toluidine blue in 1% boric acid. Ultrathin sections (70 nm thickness) were collected onto formvar-coated, slot grids, and counterstained with uranyl acetate and lead citrate. The observation and examination of the sections took place on a 100 kV Jeol JEM1230 transmission electron microscope.

### EM of Donor Eyes

Typical images of human donor eyes with and without AMD of another study^64^ were selected for this work. Healthy aged eyes without known ophthalmic diseases were obtained from the Eye Hospital Tuebingen. A glutaraldehyde-fixed tissue sample of the perimacular central region of an AMD donor eye (76 years, male) was obtained from the Foundation Fighting Blindness (United States). Written informed consent of all donors for use in medical research and additional approval of the Institutional Review Board of the University of Tuebingen were obtained. The experiments were performed in adherence to the tenets of the Declaration of Helsinki.

### VEGF-Induced Choriocapillary Remodeling in Rats

Six-week-old Long Evans rats were anesthetized and injected subretinally with 2 µL (2×10^9^ infectious units) of an adeno-associated virus (subtype 2.4) carrying the VEGF gene diluted in PBS. The vector was constructed and the research performed as described earlier for adenovirus in rabbits.^65^ Four weeks after injection, rats were euthanized and the eyes were fixed in 4% glutaraldehyde and areas showing CNV were embedded in EPON as described above for human donor eyes. The experiments were performed as approved by the Regierungspräsidium Tuebingen.

### Paraffin Sections and H&E

The fish heads were submitted to 4% formaldehyde for a minimum of 24 hours before dehydration in a series of alcohol followed by Tissue Clear (SAKURA Finetek) and embedding in paraffin (Histowax, Histolab). The paraffin-embedded fish heads were trimmed of paraffin to reach the mid part of the eye, then sectioned in 4 µm sections and mounted 2 to 3 sections/slide on Superfrost plus slides (Thermofisher). After drying for 30 minutes, the sections were stained with Hematoxylin and Eosin using Ventana Symphony staining system (ROCHE).

### Statistical Tests and Data Analysis

**Figure 1.**
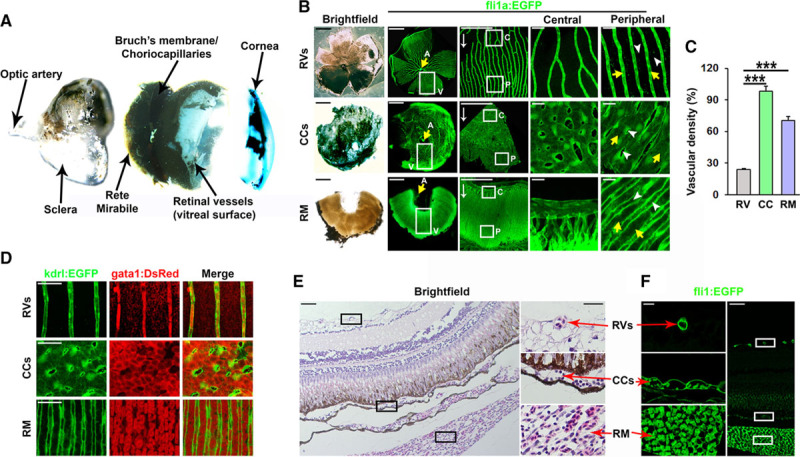
Anatomy and functional characterization of retinal vessel (RV) and choroid vessel in adult zebrafish. **A**, Bright-field micrographs showing the gross anatomy of the adult zebrafish eye. The central choroid body containing the rete mirabile (RM) is located between the choriocapillaris (CCs), which is attached to Bruch’s membrane, and the sclera. A loose retinal pigment epithelium is evident between the retina and Bruch’s membrane. The lens, located between the cornea and the retina, has been removed from the preparation. **B**, Bright-field micrographs of retinal, CC, and RM flat mounts from adult Fli1a:EGFP zebrafish and confocal micrographs of the vessels contained in these tissues (RVs, CCs, and RM, respectively). Boxed regions are shown in magnified images on the right. Central (C) and peripheral (P) vascular beds are shown separately. Yellow arrows indicate arterial branches (A) from the central optic artery, which feed blood to these vasculatures. White arrows indicate the direction of blood flow. Blood is collected by circumferential veins (V). White arrowheads in the column to the right point to interstitial spaces, whereas yellow arrowheads point to vessels/lumens. Size bars indicate 500, 500, 50, 25, or 25 μm in the first row and 100, 100, 50, 25, or 25 μm for row 2 and row 3. **C**, Quantification of the vascular density of the vasculatures shown in **B**. n=10–15. ANOVA, *P*<0.001, Tukey post hoc: ***P<0.001. **D**, Confocal micrographs of the adult RVs, CCs, and RM of kdrl:EGFP;gata1:DsRed adult double transgenic zebrafish. Endothelial cells are shown in green and erythrocytes in red. Size bars indicate 50 μm in each image. **E** and **F**, Bright-field (**E**) or confocal (**F**) micrographs of whole retina cross-sections from paraffin-embedded adult fli1a:EGFP zebrafish eyes stained with hematoxylin and eosin. The location of the RVs, CCs, and RM are shown in black or white boxes, and higher magnification images of the boxed regions are shown to the **right** or **left** in **E** or **F**, respectively. Size bars indicates 20 μm and 50 μm in the high- vs low-magnification images, respectively.

**Figure 2.**
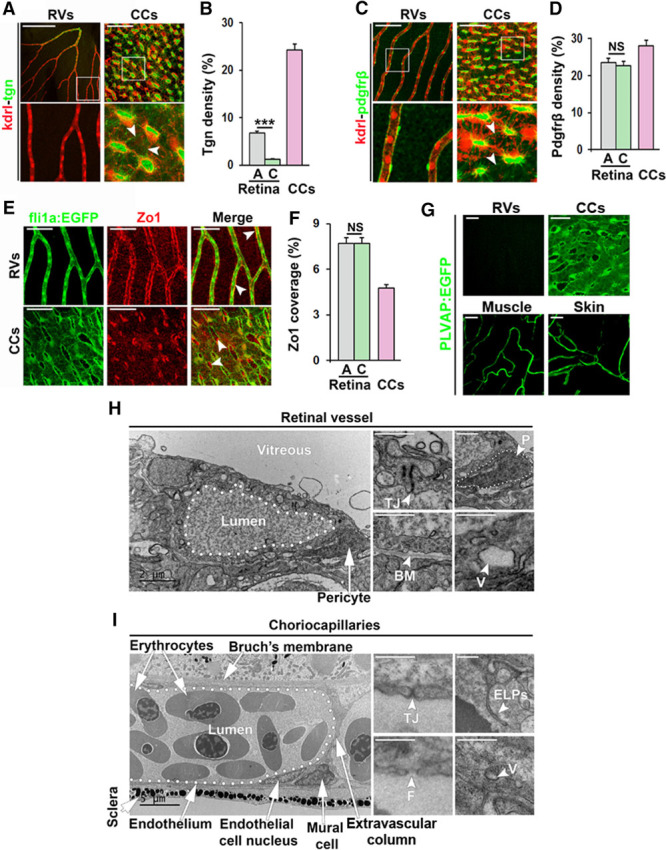
Detailed analysis of retinal vessels (RVs) and choriocapillaris (CCs) in adult zebrafish. **A** and **C**, Confocal micrographs of RVs and CCs from Kdrl:DsRed;Transgellin:EGFP (kdrl-tgn [transgellin1]) or Kdrl:DsRed;Pdgfrb:EGFP (kdrl-pdgfrb) double transgenic adult zebrafish. Endothelial cells are shown in red and smooth muscle cells (**A**) or pericytes (**C**) in green. Boxed regions are shown in the magnified images below. White arrowheads point to extensions of Tgn^+^ or Pdgfrb^+^ perivascular cell projections. Size bars indicate 100 μm for retinal vessels and 50 μm for CCs. **B** and **D**, Quantification of the percentage of the EGFP^+^ endothelium that was covered by Tgn^+^ (**B**) or Pdgfrb^+^ (**D**) cells in the experiment shown in **A** and **C**. n=5–10. ***P<0.001. **E**, Confocal micrographs of RVs or CCs from Fli1a:EGFP adult zebrafish stained with an anti–ZO (zonula occludens)-1 antibody showing endothelial cells in green and tight junctions in red. White arrowheads point to double-positive signals in the vessel wall. Size bars indicate 100 μm in retinal vessels and 50 μm in CCs, respectively. **F**, Quantification of the percentage of the EGFP+ endothelium that was stained with the anti–ZO-1 antibody in the experiment shown in **E**. n=10–15. ***P<0.001. **G**, Confocal micrographs of RVs, CCs, muscle and skin vessels from plvap:EGFP transgenic zebrafish. Cells expressing PLVAP are shown in green. Size bars indicate 50 μm. **H** and **I**, Transmission electron micrographs of RVs (**H**) or CCs (**I**). Size bars indicate 2 μm. White arrows/arrowheads point to tight junction (TJ), pericytes (P, outlined with white dots), basement membrane (BM), vesicles (V), fenestrations (F), and endothelial luminal pillars (ELPs) as indicated. A indicates arterial region; C, capillary region; and NS, nonsignificant.

**Figure 3.**
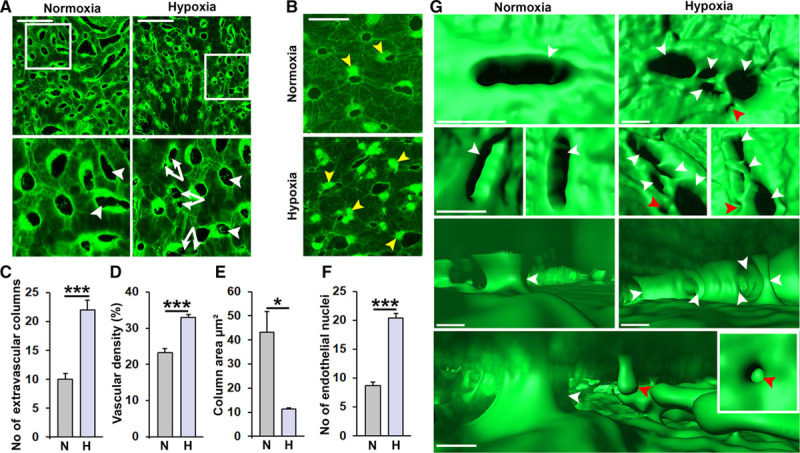
Hypoxia-induced pathological vascular remodeling in the choriocapillaris (CCs). **A** and **B**, Confocal micrographs of CCs from adult fli1a:EGFP (**A**) or fli1a:nEGFP (**B**) zebrafish exposed to water with 100% (normoxia) or 10% (hypoxia) relative air saturation for 10 d. Boxed regions are shown in **A** magnified images below. White arrowheads point to extravascular columns, double white arrows indicate split columns. Yellow arrowheads point to endothelial nuclei. Size bars indicate 50 μm. **C**, Confocal micrographs of CCs from adult fli1a:nEGFP zebrafish. Quantification of the number of extravascular columns from the experiment shown in **A**. n=10–15. ***P<0.001. **D**, Quantification of the vascular density in the CCs from the experiment shown in **A**. n=10–15. ***P<0.001. **E**, Quantification of the column area from the experiment shown in **A**. n=10–15. **P<0.01. **F**, Quantification of the number of endothelial nuclei per CC area from the experiment shown in **B**. n=10. ***P<0.001. **G**, Imaris rendering illustrating the 3-dimensional structure of the vasculature and extravascular columns viewed from outside (**top** 2 rows) or inside the CC lumen (**lower** 2 rows) from the experiment shown in **A**. Image in the lower row is from the hypoxia group. White arrowheads point to full intussusceptive pillars and red arrowheads point to incomplete pillars/endothelial luminal pillars.

**Figure 4.**
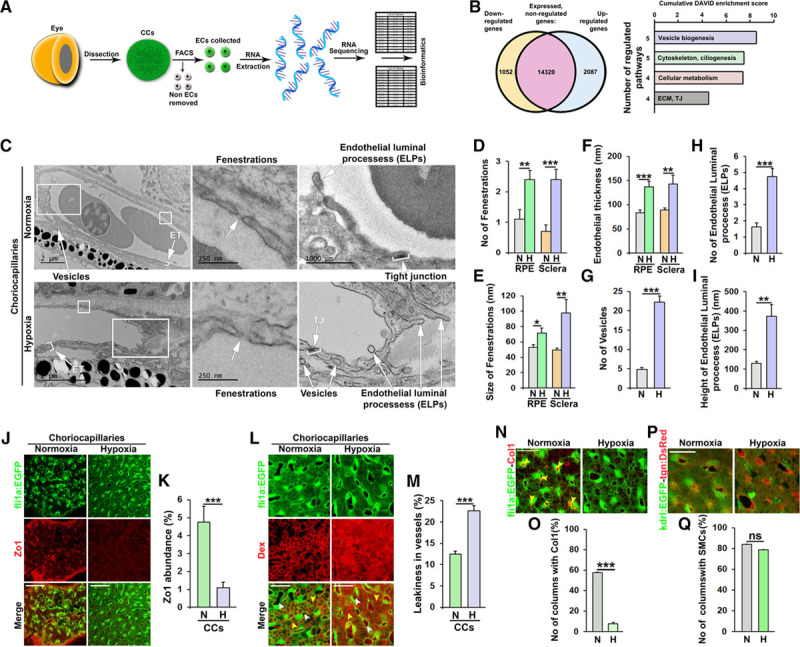
Hypoxia-induced remodeling of the choriocapillary (CC) pillars and endothelium. **A**, Schematic representation of the processes to obtain and sequence RNA from endothelial cells (ECs, shown in green) in the CCs of adult zebrafish. **B**, Schematic representation of the hypoxia-induced or hypoxia-repressed transcriptomic changes (*P*<0.05) in adult CCs ECs and families of functional annotation clusters (each with a DAVID enrichment score >1.0) in which the upregulated and downregulated genes cluster. All clusters are shown in Figure VII in the online-only Data Supplement. The numbers of clusters included in each family and the cumulative DAVID enrichment scores are indicated. **C**, Transmission electron micrographs from the CCs of adult fli1a:EGFP zebrafish exposed to water with 100% (normoxia) or 10% (hypoxia) relative air saturation for 10 d. Boxed regions are shown in magnified images at the right. White arrowheads point to endothelial thickness (ET), vesicles, fenestrations, tight junctions (TJs), or endothelial luminal processes (ELPs) as indicated. Black size bars indicate 250, 500, or 2000 nm as indicated. **D**–**I**, Quantification of the number (**D**) or size (**E**) of fenestrations and the endothelial thickness (**F**), in either the scleral or retinal pigment epithelium (RPE)-sides of the CCs, the number of vesicles (**G**) and ELPs (**H**) and the height of the ELPs (**I**) from the experiment shown in **C**. n=5–10.*P<0.05, **P<0.01, ***P<0.001. **J**, Confocal micrographs of CCs from normoxia or hypoxia-exposed adult fli1a:EGFP zebrafish (ECs shown in green) stained with an anti–ZO (zonula occludens)-1 antibody (red). Size bars indicate 50 μm. **K**, Quantification of the percentage of the EGFP+ endothelium that was stained with the anti–ZO-1 antibody in the experiment shown in **J**. n=10–15. ***P<0.001. **L**, Confocal micrographs of adult fli1a:EGFP CCs (ECs shown in green) exposed to normoxia or hypoxia for 10 d and injected with 70 kDa Dextran (Dex, red). White arrowheads point to interstitial spaces, whereas yellow arrowheads point to lumens. Size bars indicate 50 μm. **M**, Quantification of the relative percentage of interstitial dextran compared to the total signal from the experiment shown in **J**. n=15–20. ***P<0.001. **N**, Confocal micrographs of CCs from normoxia or hypoxia-exposed adult fli1a:EGFP zebrafish (ECs shown in green) stained with an anticollagen I antibody (red). Yellow arrowheads point to columns filled with collagen I–positive fibers. Size bars indicate 50 μm. **O**, Quantification of the number of columns (in percent) containing collagen I fibers in the experiment shown in **N**. n=10. ***P<0.001. **P**, Confocal micrographs of CCs from normoxia or hypoxia-exposed adult kdrl:EGFP;tgn:DsRed double transgenic zebrafish (ECs shown in green and smooth muscle cells shown in red). Size bars indicate 50 μm. **Q**, Quantification of the number of columns (in percent) containing smooth muscle cells in the experiment shown in **P**. n=10. ECM indicates extracellular matrix; H, hypoxia; N, normoxia; and ns, nonsignificant.

**Figure 5.**
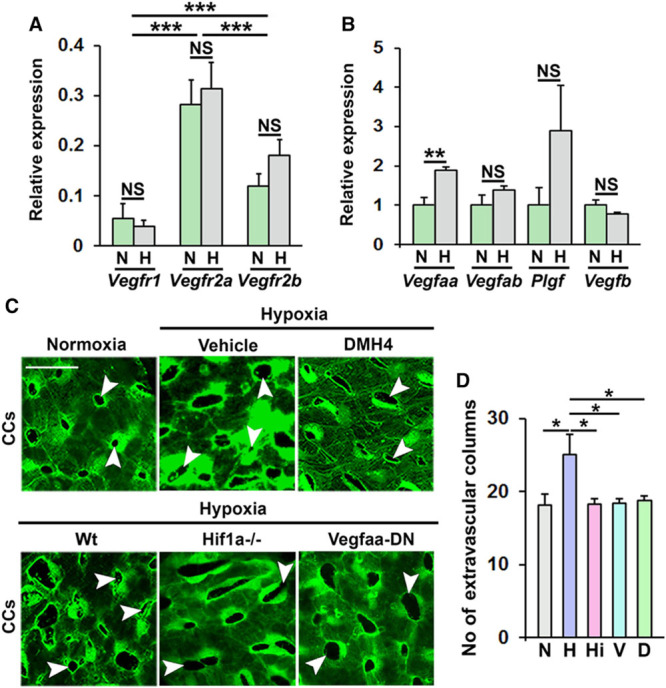
HIF (hypoxia-induced factor)-1-VEGF (vascular endothelial growth factor)-A-VEGFR2 signaling is required for hypoxia-induced pathological choriocapillary (CC) remodeling in zebrafish. **A**, Analysis of *Vegfr1*, *Vegfr2a* (*kdrl*), or *Vegfr2b* (*kdr*) mRNA expression in CCs from normoxia (N) or hypoxia (H) exposed adult fli1a:EGFP zebrafish, normalized to the expression of *Fli1a*. n=26. ***P<0.001. **B**, Quantitative PCR analysis of *Vegfaa*, *Vegfab*, *Plgf* (placental growth factor), or *Vegfb* mRNA expression in CCs from normoxia (N) or hypoxia (H) exposed adult fli1a:EGFP zebrafish, normalized to the expression of *Gapdh*. n=16–24. **P<0.01. **C**, Confocal micrographs of CCs from adult fli1a:EGFP zebrafish exposed normoxia or hypoxia and treated with DMH4, alternatively from adult hypoxia-exposed fli1a:EGFP zebrafish (Wt), fli1a:EGFP;*hif1aa*^−^^/−^;*hif1ab*^−/−^ zebrafish (HIF1a^−^^/−^), or fli1a:EGFP;Hsp70:VEGFAA-DN zebrafish incubated for 1 h at 37°C daily from day 4 of hypoxia treatment (Vegfaa-DN). White arrowheads point to hypoxia-induced extravascular columns in the CCs. Size bars indicate 25 µm. **D**, Quantification of the number of extravascular columns in the CCs from the experiment shown in **C**. N=normoxia, H=hypoxia, Hi=fli1a:EGFP;*hif1aa*^−/−^;*hif1ab*^−/−^ zebrafish, V=fli1a:EGFP;Hsp70:VEGFAA-DN zebrafish, D=DMH4-treatment zebrafish. n=10–15. ANOVA *P*<0.0053, Tukey post hoc: *P<0.05. NS indicates not significant.

The data was found to pass the D’Agostino-Pearson normality test. Differences between groups were compared by 2-tailed Student *t* test. In cases where the groups of data exhibited significantly different variance (*F* test *P* value<0.05; Figures 2B, 3E, 4G, 4I, 4K, 4M, and 4O), heteroscedastic *t* tests with Welsh correction for unequal variance was performed. For multigroup comparisons (Figures 1C and 5D), the data exhibited equal variance (*P*>0.05 using the *F* test) and significance was evaluated using ANOVA with Turkey post hoc test. *P* values of <0.05 (*), 0.01 (**), or 0.001 (***) were considered statistically significant. The data is presented as means±SEM.

## Results

### Organization of Choroid and Retinal Vessels in Adult Zebrafish

Studies into the mechanisms underlying angiogenesis in vivo are often done using the postnatal retinal vascular development model in mice.^66,67^ The retinal vasculature, however, is characterized by central nervous system–specific vascular barrier functions,^26,68^ which are not present in the choriocapillaris or other peripheral vasculatures. Investigating the physiological and molecular mechanisms involved in pathological changes in the choriocapillaris may therefore be important to gain insights into how such processes are operating in nonbarrier, peripheral vasculatures. Adult zebrafish offer unique opportunities for investigating the mechanisms underlying hypoxia-induced vascular pathology, but the zebrafish choriocapillaris has till date not been identified. Careful exploratory dissection of the adult zebrafish eye, revealed that it is highly comparable to that of mice and humans (Figure 1A through 1C) including an anterior cornea (analogous to eyelids in mammals), posterior cornea, spherical lens (as in mice), thin vitreous, retina, porous RPE, Bruch’s membrane, choriocapillaris, and outer choroidal structures including a rete mirabile (analogous to the choroid body in humans and mice) and sclera. Using fli1a:EGFP^46^ and kdrl:EGFP^47^ endothelial reporter lines, we confirmed that retinal vessels at the vitreal surface of the retina were sparse (Figure 1B and 1C; Figure I in the online-only Data Supplement) and present only in the inner limiting membrane without vessels penetrating to deeper retinal layers (Figure 1B, 1E, and 1F; Figure I in the online-only Data Supplement). We identified the zebrafish choriocapillaris, associated with Bruch’s membrane at the same location as in mammals, which compared to the retinal vasculature was extremely dense, constituting ≈95% of the tissue (Figure 1B through 1F; Figure I in the online-only Data Supplement). Vessels in the choroid body behind the choriocapillaris resembled a rete mirabile and were also exhibiting a density several-fold higher than that of the retinal vasculature at ≈70% (Figure 1B through 1F). In all 3 vasculatures (retinal vasculature, choriocapillaris, and rete mirabile), the vessels received blood from primary branches of the central optic artery and were as such organized with arteries/arterioles in the center of the optic disc followed by capillaries and draining into venules and circumferential veins at the periphery of the optic disc (Figure 1B). Unidirectional blood flow from the center to the periphery in all 3 vasculatures supported efficient perfusion, as judged from the presence of gata1:DsRed positive erythrocytes within kdrl:EGFP vessels in kdrl:EGFP;gata1:DsRed double transgenic zebrafish^48^ (Figure 1D). The differences in vascular density were confirmed by FACS-assisted counting of the number of EGFP+ ECs in either the retina or the choroid, as well as by Western blot for EGFP expression (Figure I in the online-only Data Supplement).

Consistent with central-to-peripheral organization of blood flow in the ocular vasculatures, we found using tagln:EGFP;kdrl:DsRed and acta2:EGFP;kdrl:DsRed double transgenic fish^49,50^ that all retinal vessels in the center of the optic disk were arteries densely covered with SM22-α/Tgn (transgellin1) and Acta2 (α-smooth muscle actin)-positive smooth muscle cells (SMCs), which were not present in capillaries or venous vessels (Figure 2A and 2B; Figure II in the online-only Data Supplement). Using a pdgfrb:Citrine;kdrl:DsRed double transgenic line,^56^ we found that retinal capillaries and veins were tightly covered with Pdgfrβ^+^ pericytes (Figure 2C and 2D). In contrast, Tgn^+^/Acta2^+^/Pdgfrβ^+^ SMCs were present in the interstitial space throughout the choriocapillaris and sent out thin membranous projections that only sparsely covered the endothelium (Figure 2A through 2D; Figure II in the online-only Data Supplement). To investigate molecular indicators of endothelial barrier function in more detail, we stained retinal or choriocapillaris tissues using antibodies against bona fide vascular barrier markers. GFAP-positive glial cells were densely covering retinal capillaries but were not present in the choriocapillaris (Figure IIIB in the online-only Data Supplement). Furthermore, tight junction markers ZO (zonula occludens).1 and VE-Cadherin (ie, *cdh5*) were both highly expressed in retinal capillaries but only sparsely expressed in the choriocapillaris endothelium (Figure 2E and 2F; Figure IIIA in the online-only Data Supplement). Interestingly, in the choriocapillaris, ZO.1 was robustly expressed by the SMCs (Figure 2E). To investigate whether the zebrafish choriocapillaries are fenestrated, similar to other peripheral capillary beds in for example the muscle and skin, but opposite to capillaries in the central nervous system including the retina, we evaluated the expression of the fenestration marker PLVAP (plasmalemma vesicle-associated protein; Meca32) using a newly developed transgenic reporter line.^69^ PLVAP was highly expressed in the choriocapillaris, as well as in capillaries of the muscle and skin, but not in the retinal endothelium of adult zebrafish (Figure 2G).

Consistent with the histological results, TEM analysis revealed that zebrafish retinal capillaries were richly covered with pericytes and a continuous basement membrane shared by both pericytes and ECs. Tight junctions were clearly apparent at EC-EC cell junctions, and the endothelium contained numerous vesicles indicative of active transport of substances across the blood-retinal barrier (Figure 2G). No fenestrations could be observed in the retinal capillary endothelium. On the contrary, choriocapillaries were sparsely associated with SMCs and basement membrane, exclusively at the scleral side. The retinal side was instead closely associated with Bruch’s membrane, fenestrated and contained only few, small vesicles as the endothelium was very thin. Interendothelial tight junctions were present but short and poorly developed compared to in the retina (Figure 2H). Taken together, these findings demonstrate that the choriocapillaris in adult zebrafish are highly similar to choriocapillaris in humans, and that they are comparable to peripheral capillaries, whereas retinal vessels are comparable to retinal and brain vessels in humans and both functionally and morphologically different from choriocapillaris and peripheral capillaries. Interestingly, a few endothelial luminal projections (ELPs; Figure 2G and 2H) could occasionally be observed in the adult zebrafish choriocapillaris, structures that may represent early stage ISPs suggesting that physiological, continuous remodeling of the choriocapillaris occurs through intussusception.

### Hypoxia Induces Pathological Choriocapillaris Remodeling by Intussusception

Hypoxia is considered a main pathophysiological driver of neovascularization in both health and disease.^30,70^ To investigate if hypoxia treatment affects adult zebrafish choriocapillaris, fli1a:EGFP zebrafish were placed in water which was rendered hypoxic through perfusion of N_2_ gas until reaching a level of 10% relative air saturation (ie, ≈2% oxygen), which was then maintained for 10 days.^33,34,42^ Hypoxia-treated zebrafish were then compared to control animals exposed to 100% relative air-saturated water. Surprisingly, 10 days of hypoxia treatment did not lead to an increase in choriocapillaris sprouting (Figure 3A), unlike what had been previously observed in the retinal vasculature,^33,34^ nor did hypoxia induce vascular invasion through Bruch’s membrane and into the subretinal space. However, a robust induction of additional, circular transluminal, extravascular columns through the choriocapillaris was observed in hypoxia-treated compared to control zebrafish, and these columns had a reduced mean area leading to increased overall vascular density (Figure 3A through 3E). Furthermore, many of the columns in hypoxia-treated fish seemed to be in close contact with each other, separated only by a thin endothelial membrane (Figure 3A; white double arrows). In addition, hypoxia treatment induced a pronounced proliferation of choriocapillaris ECs, as indicated by condensation and duplication of nuclear-restricted EGFP within ECs as well as an increase of total EC nuclei (Figure 3B and 3F). To validate that the zebrafish eyes were indeed hypoxic in the hypoxia-treated fish, we strained retinal cross-sections with antibodies recognizing HIF-1a or HIF-2a, proteins that should be unstable and therefore not detectable under normoxic conditions. In the outer nuclear layer, the most metabolically active area in the retina, of hypoxia-treated fish, HIF-1a and HIF-2a immune-positivity was markedly elevated (Figure IV in the online-only Data Supplement), demonstrating that these proteins were indeed stabilized by the hypoxia treatment.

Elevated blood pressure, leading to increased mechanical stimulation of the endothelium, affects vascular growth and remodeling.^71^ As hypoxia treatment is known to cause blood pressure elevation in zebrafish,^42^ we tested whether increased blood pressure by itself would affect the choriocapillaris in a similar manner as in hypoxia-treated fish. Increasing the blood pressure by treatment with the β-adrenergic receptor agonist Clenbuterol for 10 days did not, however, lead to an increase in ISPs, and the choriocapillaris remained indistinguishable from those in control fish, suggesting that hypertension alone did not cause changes to the choriocapillaris in hypoxia-treated zebrafish (Figure V in the online-only Data Supplement).

To identify the structural processes underlying hypoxia-induced intussusception in the zebrafish choriocapillaris, we analyzed the 3-dimensional (3D) structure of the extravascular columns and the intravascular lumens of control and hypoxia-treated fish using Imaris rendering of high-power 3D-confocal z-stacks. Interestingly, numerous incomplete extravascular columns were apparent in the intravascular lumen of hypoxia-treated but not in control fish (Figure 3G; red arrowheads), providing further evidence for ongoing hypoxia-induced intussusception. In addition, we also found that existing extravascular columns were split to generate multiple, smaller, closely associated columns specifically in the hypoxia-treated fish (Figure 3G). Such splitting of extravascular columns appeared to involve active cleavage of the columns by EC processes that expanded from one side of the column to anastomose with a projection or endothelial surface at the opposing side. These processes quickly became lumenized to support perfusion around the now 2 (or more) separate extravascular columns (Figure 3G). As such, hypoxia-induced intussusception occurred both by de novo ISP formation and by splitting of existing extravascular columns.

Next, we investigated the molecular mechanisms underlying hypoxia-induced intussusception in the choriocapillaris by sequencing RNA isolated from EGFP+ ECs collected by FACS from hypoxia-treated or control zebrafish choriocapillaris membranes (Figure 4A). As expected, EC populations expressed high levels of classical EC markers including *fli1a*, *fli1b*, *flt1*, *kdr*, *tie1*, and *tek* (Table II in the online-only Data Supplement), whereas genes associated with other vascular or choroidal cell types such as neurons (*dbx1*, *dbx2*), glia cells (*gfap*, *aldh1a3*, *mbp*, *pax6a*, *pax6b*, *calb2*), SMCs (*desm*, *abcc9*, *kcnj8*), or pericytes (*cspg4*, *desm*) were not significantly expressed (ie, RPKM [reads per kilobase million] <5; Table III in the online-only Data Supplement), confirming that we had obtained a highly EC enriched population of cells. Only 3 genes, *hbz*, *hbbe2*, and *ccl34a.4* (*si:ch211-122l24.4*), were significantly deregulated (induced) in the hypoxia-treated fish using a false discovery rate <0.05 (Table IV in the online-only Data Supplement). These genes encode 2 hemoglobin isotypes and a protein known to be involved in hematopoiesis, all 3 genes being specific for erythroid cells^72^ and therefore likely expressed in circulating fli1a-positive hematopoietic progenitor cells present in the choriocapillaris at the time of cell isolation, rather than in the vascular EC population. Nevertheless, these findings demonstrate that the fish were exhibiting expected physiological adaptations to long-term hypoxia such as increased erythropoiesis. Using instead a less rigorous cutoff (*P*<0.05), a total of 2087 upregulated and 1052 downregulated transcripts (Figure 3E; Table IV in the online-only Data Supplement) were identified. Using an in house algorithm^73^ to analyze these genes for the presence of hypoxia-responsive elements in their promoters, we could demonstrate that directly HIF-responsive genes were not enriched in this set of genes (Figure VI in the online-only Data Supplement). This likely implies that the expression of the majority of deregulated genes was controlled by hypoxia-regulated transcription factors rather than the HIFs themselves, under these experimental conditions. Using the DAVID functional annotation tool, we found that the majority of the deregulated genes clustered into a total of 13 upregulated and 9 downregulated pathways (Figure VII in the online-only Data Supplement). Interestingly, 18 of these 22 pathways had very similar biological functions and could be assembled into 4 pathway families (Figure 4B). These 4 families included pathways involved in vesicle biogenesis, cytoskeletal rearrangements and ciliogenesis, regulation of cellular metabolism and ECM (extracellular matrix) or tight junction remodeling. Changes in cellular metabolism are expected in response to hypoxia, but the remaining 3 pathway families have not been strongly coupled to hypoxia or hypoxia-induced vascular growth or remodeling in the past. Using phalloidin staining and a fli1a:GFF;UAS:RFP;UAS:UCHD-EGFP triple transgenic zebrafish strain^55^ in which an EGFP-fused utrophin calponin homology domain protein, binding specifically to f-actin, was expressed in ECs, extravascular pillars, and ELPs indicative of early ISPs were found to be strongly enriched for f-actin, which was not observed in the endothelium outside of these structures (Figures VIII and IX in the online-only Data Supplement). These findings support the involvement of the actin cytoskeleton in the observed hypoxia-induced intussusception in the choriocapillaris. Taken together these results strongly suggest that hypoxia may drive intussusceptive choriocapillaris growth through a mechanism involving membrane and cytoskeletal rearrangements in ECs.

### Hypoxia Treatment Drives Nonproductive Intussusception and Leakage in the Choriocapillaris

To further analyze how pathways involved in vesicle biogenesis, cytoskeletal dynamics/ciliogenesis, and ECM/tight junction remodeling are involved during hypoxia-induced intussusception and vascular remodeling, we performed TEM analysis on the choriocapillaris from control or hypoxia-treated zebrafish. We found that hypoxia-treated zebrafish had an increased number and size of endothelial fenestrations (Figure 4C through 4E; Figure X in the online-only Data Supplement) and a thickened endothelium (Figure 4C and 4F) in both the RPE- and scleral sides of the vasculature. Furthermore, we found a dramatic induction of endothelial vesiculation, with almost 6-fold more vesicles present in the endothelium of hypoxia-treated fish compared to normoxia controls (Figure 4C and 4G). Interestingly, we also found a dramatically elevated number and size of ELPs, which often did not proceed to form mature intussusceptive transluminal pillars (ISPs; Figure 4C, 4H, and 4I). Often, a markedly thickened and heavily vesiculated endothelium coincided with ELP formation leading to congested lumens in these areas (Figure 4C; Figure X in the online-only Data Supplement). Such areas were never found in control fish. We did not find any evidence of perivascular cell processes in the ELPs, which consisted only of luminal sprouts from the ECs. As such, ELPs could possibly be considered large endothelial cilia and their formation likely controlled by the ciliogenesis pathways upregulated during hypoxia treatment. Interestingly, we found that tight junctions were partially dissolved in the choriocapillaris of hypoxia-treated fish (Figure 4C), which was confirmed by a reduction in ZO.1 expression in both endothelial and SMCs (Figure 4J and 4K). This phenotype was also supported by the transcriptomic data demonstrating that pathways involved in tight junction remodeling were affected by the hypoxia treatment (Figure 4B). Importantly, these pathological changes led to a prominent increase in leakage of rhodamine-conjugated, lysine-modified 70 kDa Dextran (Figure 4L and 4M). Furthermore, areas in which the endothelium was open, indicative of previous or ongoing hemorrhaging was present in several hypoxia-treated zebrafish, but never in controls (Figure X in the online-only Data Supplement). These areas were often associated with a blot clot, as expected, but also a dramatically elevated number of ELPs, which presumably helped to constrict the lumen, reduce the bleeding, and heal the injury.

Next, we analyzed the composition of the extravascular columns in control or hypoxia-treated zebrafish. TEM analysis revealed that while collagen fibers were clearly present in these columns under normoxic conditions, no such fibers could be detected in the columns of hypoxia-treated zebrafish (Figure XI in the online-only Data Supplement). Furthermore, while SMCs were still present in the columns under hypoxic conditions, the normally very tight association between the SMCs and the endothelium found under normoxic conditions, was disrupted in hypoxia-treated zebrafish resulting in a loose association between the SMCs and ECs (Figure XI in the online-only Data Supplement). These findings were validated by immunohistological characterization of collagen I content in the choriocapillaris. While collagen I–positive fibers were filling most of the extravascular columns in normoxic fish, such fibers were completely absent in hypoxia-treated fish (Figure 4N and 4O), whereas overall SMC investment was not changed (Figure 4P and 4Q).

Collectively, these results demonstrate that signaling pathways involved in vesicle biogenesis, cytoskeletal dynamics, ciliogenesis, and tight junction remodeling coupled to a degradation of collagen fibers and a loosening of EC-SMC contacts are functionally linked to hypoxia-induced PVR.

### HIF-1α-VEGFA-VEGFR2 Signaling Is Critical for Hypoxia-Induced PVR in Zebrafish

One of the main hypoxia-induced factors mediating pathological vascular phenotypes in disease is VEGF-A.^74^ Using quantitative PCR we investigated the expression of VEGF-ligands and receptors in whole choriocapillaris membranes from control and hypoxia-treated zebrafish and found that *Vegfaa*, *Vegfab*, *Vegfb*, *Plgf* (placental growth factor), *Vegfr1*, *Vegfr2a*, and *Vegfr2b*, mRNAs were expressed in both hypoxia-treated and control zebrafish. Only *Vegfaa*, coding for the primary zebrafish VEGF-A ligand was, however, upregulated in hypoxia-treated fish (Figure 5A and 5B). Furthermore, *Vegfr2a* (*Kdrl*) coding for the main VEGF-receptor was expressed at higher levels compared to the other receptors (Figure 5A). Using immunohistochemistry, we found that phosphorylation of VEGFR2 was increased in both retinal vessels and choriocapillaris of hypoxic fish, and that known VEGF-induced or VEGF-repressed genes^75–80^ were similarly regulated in the ECs from the choriocapillaris of hypoxia-treated fish compared to normoxic controls (Figure XII in the online-only Data Supplement).

Next, using newly developed genetic loss-of-function models including a HIF-1αa/HIF-1αb-double knockout strain,^57^ a heat-shock inducible VEGFA-dominant negative strain,^58^ and a specific inhibitor of VEGFR2 (when used at doses that does not inhibit VEGFR1-signaling^79,80^), we found that hypoxia-induced intussusception was completely blocked under these 3 conditions (Figure 5C and 5D). Combined these results demonstrate that hypoxia-induced intussusception in zebrafish occurred through the HIF-1α-VEGFA-VEGFR2 pathway.

### Productive and Nonproductive Intussusception Is Associated With PVR in Wet AMD

**Figure 6.**
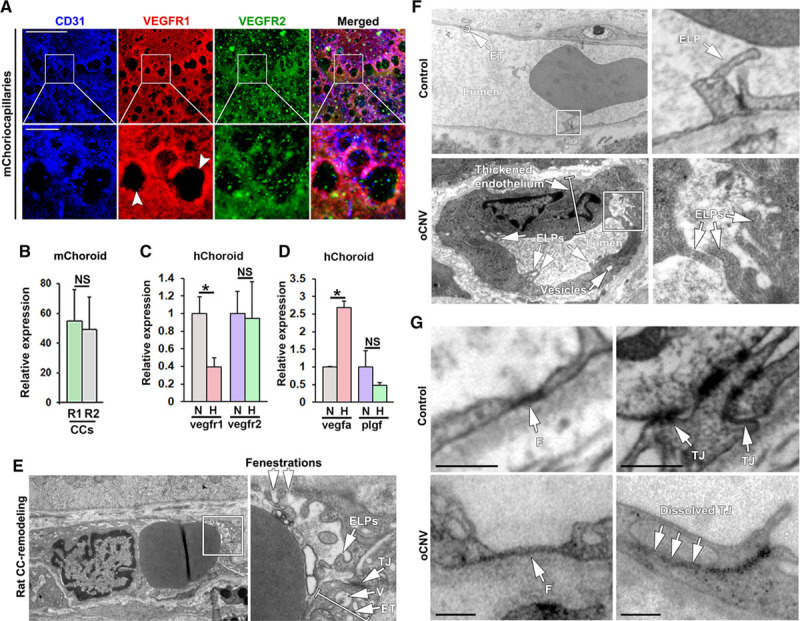
Pathophysiological changes in the choriocapillaris (CCs) in adult zebrafish are recapitulated in mice, rats, and human wet age-related macular degeneration (AMD) patients. **A**, Confocal micrographs of CCs from adult C57/Bl6 mice (m) stained with anti-CD31 (blue), anti-VEGFR1 (red), and anti-VEGFR2 (green) antibodies. Boxed regions are shown in the magnified images below. White arrowheads indicate interstitial areas/extravascular columns. Size bars indicate 100 µm for the **top** row and 20 µm for the **lower** row. **B**, Quantifications of the percentage of the CD31+ endothelium that was costained with the VEGFR (R1) and VEGFR (R2) antibody in the experiment shown in **A**. n=3. **C**, Quantitative PCR analysis of *Vegfr1* and *Vegfr2* mRNA expression in primary human choroidal endothelial cells (hChoroid) in culture following 12 h of treatment with normal oxygen (normoxia, N) or 1% oxygen (hypoxia, H), normalized to the expression of TATA box-binding protein. n=3. *P<0.05. **D**, Quantitative PCR analysis of *Vegfa* or *Plgf* expression in primary human choroidal ECs (hChoroid) subjected to normoxia or hypoxia as in **C**. n=3. *P<0.05, ***P<0.001. **E**, Transmission electron micrographs of rat CC remodeling following adeno-associated virus-mediated VEGF (vascular endothelial growth factor)-A overexpression in the outer retina/subretinal space. Boxed region is shown in the magnified image to the **right**. White arrows point to endothelial luminal processes (ELPs), tight junctions (TJs), vesicles (V), endothelial thickness (ET), and fenestrations, as indicated. **F**, Transmission electron micrographs of human occult choroidal neovascularization (CNV) or non-CNV control CCs. Boxed regions are shown in magnified images to the **right**. White arrows point to ELPs and ET. Size bars indicate 2 μm or 1 μm, respectively. **G**, Transmission electron micrographs of details from human occult choroidal neovascularization (oCNV) or non-CNV control choriocapillaris. White arrows point to fenestrations (F) and TJs, as indicated. Size bars indicate 1 μm. NS indicates not significant.

Next, we investigated the expression of VEGF-receptors in adult mouse choriocapillaris by immunohistochemistry. Both VEGFR1 and VEGFR2 were strongly expressed by the choriocapillaris endothelium (Figure 6A and 6B). To investigate if this was also the case in humans, we purified ECs from the choroid of a donor with no history of ocular disease.^63^ These ECs were then hypoxia-treated to simulate the pathological condition in the zebrafish model. Also in human choroid ECs, hypoxia treatment produced a VEGFR2-biased signaling cascade by downregulating VEGFR1, considered to be a negative inhibitor of VEGFR2 signaling,^60^ and upregulating VEGFA, while neither significantly changing the expression levels of VEGFR2 itself nor the VEGFR1-only ligand PlGF (Figure 6C and 6D). To further demonstrate that VEGFA signaling may lead to the phenotypes observed in the zebrafish, we took advantage of a gain-of-function adeno-associated virus delivery system to overexpress VEGFA in the rat choroid. PVR in adeno-associated virus-VEGFA-treated rats was investigated by TEM. This analysis clearly demonstrated that VEGFA-conditioned choriocapillaris developed numerous, enlarged fenestrations, disrupted tight junctions, thickened and highly vesiculated endothelium, a high number of ELPs and occasionally open areas of the endothelium, thus accurately phenocopying the PVR observed in hypoxia-treated zebrafish (Figure 6E; Figure IX in the online-only Data Supplement).

To further translate the role of functional and nonfunctional intussusception in PVR associated with CNV, we analyzed choriocapillaris from AMD patients and non-AMD controls by TEM. As observed during hypoxia-induced PVR in zebrafish and VEGF-A–induced PVR in rats, the pathologically activated, but not neovascular choriocapillaris in AMD patients, were characterized by a large number of ELPs, a few of which connected with ELPs from the opposing side to form primitive ISPs, suggesting that productive and nonproductive intussusception is also important for PVR in humans (Figure 6F). Furthermore, the endothelium was markedly thickened and vesiculated, tight junctions were partially dissolved and enlarged fenestrations were present (Figure 6F and 6G), phenotypes closely resembling those found in the zebrafish and rat models.

**Figure 7.**
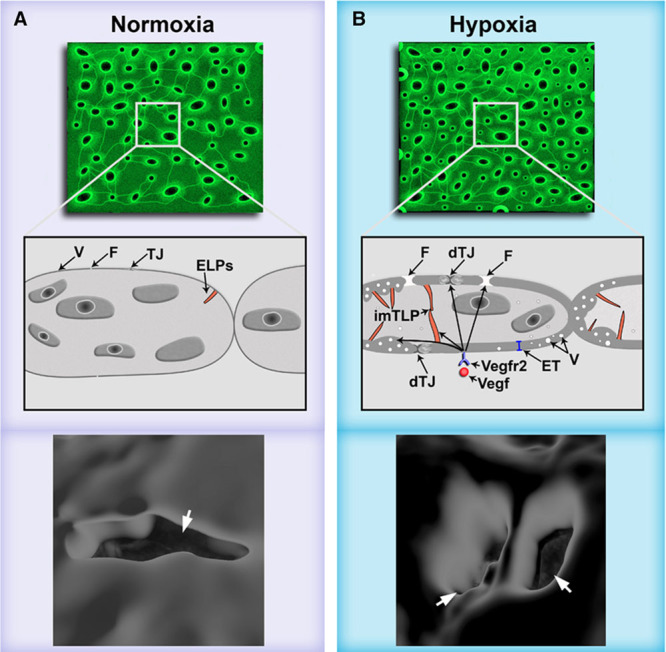
Scheme illustrating the proposed mechanisms underlying mechanisms involved in pathological choriocapillary (CC) remodeling preceding choroidal neovascularization (CNV). **A**, Illustration of healthy, normoxic choriocapillaries. **B**, Hypoxia-induced pathological CC remodeling is driven by HIF (hypoxia-induced factor)-1-VEGF (vascular endothelial growth factor)-A-VEGFR2-induced cytoskeletal dynamics/ciliogenesis, vesicle biogenesis, tight junction (TJ), and extracellular matrix remodeling pathways, leading to (1) vascular leakage through dissolved TJs (dTJs) and enlarged fenestrations (F), (2) productive and nonproductive intussusception including formation of endothelial vesicles (V), increased endothelial thickness (ET), and endothelial luminal projections (ELPs) some of which connected to ELPs from the other side of the endothelium leading to immature intussusceptive pillars (imISPs), and (3) splitting of extravascular columns by endothelial processes extending across a column (indicated by white arrowheads) to generate 2 new, smaller columns.

In summary, the data presented here reveal a novel type of PVR that precede sprouting and neovascularization in the choriocapillaris of hypoxia-treated adult zebrafish, VEGFA-treated rats and in AMD patients. Mechanistically, PVR was mediated by HIF-1-VEGFA-VEGFR2 signaling, and involved intussusception associated with the local congestion of choriocapillaris lumens through endothelial thickening, vesiculation, and ELP formation (collectively referred to here as nonproductive intussusception) as well as productive splitting of existing extravascular columns by degradation of their collagen core followed by extension of endothelial processes through the columns. In addition, dissolved tight junctions and enlarged fenestrations in hypoxia-treated fish mediated vascular leakage and likely drive disease progression in AMD at an earlier stage, before CNV (summarized in Figure 7).

## Discussion

Inhibiting or reversing pathological neovascularization has emerged as an important concept in treatment of a variety of disorders including cancer and eye diseases such as AMD and DR.^2,12,25^ The anti-VEGF drugs in clinical use today are offered to patients diagnosed with advanced, neovascular disease stages with the goal to achieve regression of the pathological, ectopic vasculature present in the tissue thus avoiding further damage due to vascular dysfunction.^6,8,10^ These treatments often provide short-term therapeutic benefit due to initial regression of immature neovessels, but most patients sooner or later develop refractory disease characterized by resumed pathological neovascularization arising from the matured neovessels that remain after anti-VEGF treatment in the diseased tissue. As such, many patients eventually progress in spite of continued treatment.^12^ As anti-VEGF drugs can be toxic when given systemically and for long periods of time, treatment schedules often include drug holidays, which, however, may be associated with worsened prognosis of some patients, presumably due to rebound neovascularization in the treatment-free periods.^83,84^ It is likely that if antineovascular drugs are given at early disease stages, where pathological vascular activation and remodeling has not yet progressed to ectopic neovascularization, they could potentially prevent progression to advanced disease, and provide more effective, long-term disease control. Methods to identify early changes in blood vessels preceding neovascularization are however lacking as the underlying mechanisms are poorly understood. Whereas some diseases such as cancer have already progressed to neovascular stages on diagnosis, AMD patients are often diagnosed at preneovascular stages, and regularly monitored.^9^ As such, given a robust clinical indication of initial vascular changes preceding neovascularization, there is a real chance that this could translate to earlier treatment aiming at preventing progression to advanced disease and retained vision in AMD patients. Clinically, the PVR phenotypes discovered here would likely imply changes in blood flow or leakage that could be evaluated today using modern imaging techniques such as fluorescein and optical coherence tomography angiography.^85^ Examinations based on these techniques could potentially be developed and used as a clinical readout for increased risk of developing neovascular disease, which could indicate the use of preventive antineovascular drugs in the future.

Using a novel adult zebrafish model whereby the long-term effects of hypoxia on the choriocapillaris could be studied, we delineated a previously unidentified PVR and leakage mechanism in the choriocapillaris, which was evident before signs of CNV and involved increased endothelial fenestration, dissolution of tight junctions, endothelial vesiculation and thickening, and productive and nonproductive intussusception. Likely due to the extremely high density of the choriocapillaris at baseline,^86^ we found that splitting of extravascular columns by fusion of endothelial processes from ECs on opposing sides of the column, coinciding with robust endothelial proliferation, played a major role in further increasing vascular density in the choriocapillaris following hypoxia treatment. We further demonstrate that these phenotypes also dominate PVR associated with choriocapillaris vascular lesions that had not yet penetrated through Bruch’s membrane in rats and humans. Vascular remodeling and leakage were transcriptionally linked to pathways involved in cytoskeleton remodeling and ciliogenesis, vesicle biogenesis, ECM, and tight junction remodeling. These pathways converged to drive productive and nonproductive intussusception, associated with dramatically increased vesiculation and thickening of the endothelium. Previously, vascular remodeling in the choriocapillaris has been found to, in extreme cases, lead to occlusion of >90% of the lumen through the formation of labyrinth-like vascular structures in wet AMD patients,^87^ suggesting that luminal occlusion and disruption of blood flow by local accumulation of ELPs as shown here, are likely highly relevant for and associated with human neovascular disease.

Intussusception has primarily been considered as an alternative to sprouting angiogenesis and a mechanism of vascular maturation and remodeling of newly developed vascular networks.^14,17,19,21^ Based on the PVR mechanisms presented here, we propose that intussusception may also be a vascular priming step that precedes neovascularization. Intussusception in this aspect was characterized by robust EC proliferation and degradation of the collagen content in existing extravascular columns, followed by column splitting as well as nonproductive intussusception, that is, the generation of ELPs that to a large extent failed to mature into complete ISPs. Several cellular and molecular pathways leading to intussusception and formation of mature ISPs have been considered.^13,88^ In tumors, for example, intussusception has been suggested to occur by f-actin–mediated pulling of extracellular but EC-associated collagen I bundles through the pillar, as it forms.^16^ Conversely, during development, it has been reported that the process is facilitated by myofibroblasts pushing through the endothelium from the basolateral side towards the lumen, thereby creating the pillar.^88^ In support of a stabilizing role of perivascular myofibroblasts/SMCs, we find that αSMA^+^/Transgellin^+^/Pdgfrβ^+^ SMCs are localized at perivascular positions in the choriocapillaris, with their cell bodies inside the extravascular columns, stretching thin processes across the endothelium presumably as a means of providing trophic support. While the SMC investment was similar in hypoxia-treated fish, the SMC-EC connections were, however, significantly loosened during the PVR process. In addition, TEM and immunohistochemistry clearly indicated that mature extravascular columns contained collagen I fibers, but that these unlike the SMC content of the columns, are degraded following hypoxia treatment, likely as an important step in the vascular fusion/column splitting process. The transcriptomic and TEM data presented here also suggests that intussusception, at least initially, is an EC-orchestrated phenomenon in which the formation of endothelial processes from the luminal side drives early pillar formation in a manner that does not involve changes to the basolateral side of the endothelium, and therefore is not dependent on perivascular collagen I fibers, myofibroblasts or other nonendothelial structures or cells. Indeed, our data clearly show that the basolateral endothelial membrane is smooth and maintain a similar structure at sites of ELP formation, as at other sites.

The use of zebrafish to understand molecular mechanisms involved in vascular pathology and hypoxia benefits not only from the fact that pathologically relevant levels of hypoxia can be induced by environmental hypoxia treatment as shown in this work, but also because HIF-1α knockout zebrafish are viable.^57^ HIF-1α knockout mice die in utero due to an inhibition of developmental angiogenesis.^89^ Zebrafish embryonic tissues, on the contrary, are oxygenated by passive diffusion of oxygen from the water, and developmental angiogenesis is therefore not hypoxia- or HIF-1–dependent in this organism.^60,90^ This allows embryos to develop in the absence of HIF-1α and survive to adulthood. This unique animal model for complete lack of HIF-1 signaling^57^ now provides an opportunity to understand HIF-1–dependent and independent mechanisms of hypoxia-induced PVR and angiogenesis.

VEGFA-VEGFR2 signaling has traditionally been considered the main mechanism underlying hypoxia-/HIF-1–induced vascular responses including angiogenesis and vascular destabilization, whereas VEGFR1 in many cases inhibit angiogenesis by acting as a VEGF-A scavenger.^91^ Similarly, PlGF, which does not bind to VEGFR2, form heterodimers with VEGFA and therefore inhibit VEGFR2-signaling, at least when PlGF and VEGFA are produced by the same cell.^92^ We demonstrate that in human choroidal ECs, PlGF, and VEGFR1 are downregulated in response to hypoxia, whereas VEGF-A is dramatically upregulated, findings that were confirmed in the zebrafish hypoxia model, suggesting that hypoxia leads to a switch towards VEGF-A-VEGFR2 signaling in choroidal ECs. This was likely HIF-1α-dependent as we found a complete lack of hypoxia-induced intussusception in HIF-1α-KO zebrafish. While in this work we focus on the role of VEGF-A-VEGFR2 signaling, it is likely that other hypoxia-induced factors also play a role during PVR.^12,25^ In our genome-wide analysis of hypoxia-induced changes to the endothelial transcriptome, we find that many receptors for secreted factors with potential roles in neovascularization were upregulated in ECs from hypoxia-treated zebrafish, although not to a significant extent following correction for multiple testing (false discovery rate >0.05). The role of VEGFA-VEGFR2 during PVR preceding neovascularization described here does therefore not preclude the possibility that other factors could also be involved in this process. Indeed, VEGFA-VEGFR2 signaling is also important for neovascularization in most other disease models including postnatal retinal vascular development^93^ and tumor angiogenesis,^40,94^ but these models are still today important and heavily used tools for discovering alternative non-VEGF mechanisms, which also play important roles in pathological vessel growth. We expect that the zebrafish hypoxia-induced pathological choriocapillaris remodeling model presented here and now validated in terms of VEGF-VEGFR2 signaling will similarly prove valuable for identifying and mechanistically linking other factors and targets of clinical importance to this process in the future.

In conclusion, we have here demonstrated that pathological neovascularization is preceded by vascular remodeling, which occurs through productive and nonproductive intussusception due to VEGF-VEGFR2–induced formation of ELPs and increased endothelial fenestration, thickening, vesiculation, and disruption of tight junctions. These findings shed new light on the process of intussusception, indicating that this is initially regulated mainly by luminal endothelial sprouting without involvement of basolateral or perivascular structures or cells, and suggest a role for intussusception in endothelial priming before neovascularization.

## Acknowledgments

We thank the Linköping University zebrafish facility technical staff for outstanding service. We also like to extend our deepest gratitude to the donors and their families for donating the eye tissues, the Cornea Bank of the University Eye Hospital Tuebingen, and the Foundation Fighting Blindness for providing the tissue samples. We thank Didier Stainier (DYRS), Oliver Stone, and Andrea Rossi for sharing invaluable zebrafish strains and fruitful discussions relating to the article. C. Gerri and M. Marass thank DYRS (MPIHL) for funding and support. We thank Emma Lardner for technical support. L.D. Jensen, J. Kele, Y. Cao, N. Lagali, J.F. Steffensen, H. Andre, M. Ntzouni, and B. Peebo designed research; Z. Ali, L.D. Jensen, J. Kele, A. Mukwaya, A. Biesemeier, M. Ntzouni, D. Ramsköld, S. Giatrellis, P. Mammadzada, R. Cao, A. Lennikov, C. Hildesjö, and Q. Deng performed research; Z. Ali, A. Mukwaya, L. del Peso, Y. Cao, A. Biesemeier, J. Kele, and L.D. Jensen analyzed data; M. Marass, C. Gerri, M. Taylor, L. del Peso, U. Schraermeyer, A. Kvanta, H. Andre, and R. Sandberg contributed vital new reagents or analytical tools; and L.D. Jensen wrote the article with input from all the authors.

## Sources of Funding

This study is supported by The Crown Princess Margareta Association for the Visually Impaired, Edwin Jordan Foundation, The Swedish Eye Foundation, Svenska Sällskapet för Medicinsk Forskning, Linköping Universitet, Eva och Oscar Ahréns Stiftelse, Ollie och Elof Ericssons Stiftelse, Carmen och Bertil Ragnérs Stiftelse, Gösta Fraenkels Stiftelse, Åke Wibergs Stiftelse, Lions Forskningsfond, Karin Sandbergs Stiftelse, Cancerfonden, and Karolinska Institutets Stiftelser och Fonder and Vetenskapsrådet.

## Disclosures

None.

## Supplementary Material
