# Supplementary material for: Intussusceptive Vascular Remodeling Precedes Pathological Neovascularization
Source: Arterioscler Thromb Vasc Biol. 2019 May 9;39(7):1402–18. doi: 10.1161/ATVBAHA.118.312190 (PMC6636809; doi:10.1161/ATVBAHA.118.312190)

Supplemental information

Additional, non-essential details to materials and methods

Hypoxia exposure.

Water oxygen levels were controlled by an oxygen regulator (Loligo Systems, Denmark), which engaged/disengaged the perfusion of nitrogen into the water depending on the oxygen concentration, measured by an in-tank oxygen electrode. The water was constantly stirred to avoid buildup of oxygen gradients, and was covered by a loose lid.

Zebrafish euthanasia and dissection.

Dissection of the eyes was performed with the aid of tweezers (Dumont # 5) and spring scissors (Agnthos AB, Sweden) to cut out the cornea and remove the retina. The multilayered choroid body/rete mirabile was slowly removed from the CC, which was peeled off together with Bruch's membrane from its attachment to the retinal pigment epithelium. The isolated retina was then cut 4-5 times around its wall and flat mounted on a glass slide. The CC was flat mounted without incisions. Tissues were mounted in Vectashield (H-1000 Vector laboratories) within a viewing chamber on a glass slide. The top cover slide was separated from the glass slide by spacers (1 cover-slide high) and fixed in place by nail polish.

RNA-sequencing

Reads were aligned with STAR to the genome (assembly danRer6, because of annotation issues with danRer7). Non-uniquely aligning reads were discarded. We then calculated expression values and read counts using rpkmforgenes (available at

http://sandberg.cmb.ki.se/rnaseq/) with settings -fulltranscript -mRNAnorm -mrnameoverlap -bothendsceil and Ensembl gene annotation from 20 Aug 2010. As quality control, we calculated Spearman correlation between samples, and removed the worst samples. All samples within each group were comparable after multiple testing correction. Raw data and expression values have been deposited to NCBI Sequence Read Archive with ID SRP056125 and Gene Expression Omnibus with ID GSE66848. False discovery rates were calculated by the Benjamini-Hochberg method.

Human choroidal and retinal qPCR analysis.

The primer sets were purchased from Eurogentec (BioNordika Sweden AB) and sequences are listed as follows: hVEGFA-forward: 5’-AGGGCA-GAA-TCA-TCA-CGA-AGT-3’ and -reverse: 5’-AGG-GTC-TCG-ATT-GGA-TGG 4 CA-3’. hPlGF-forward: 5’-GAA-CGG-CTC-GTC-AGA-GGT-G-3’ and -reverse: 5’-ACAGTG-CAG-ATT-CTC-ATC-GCC-3’. hVEGFR1-forward: 5’- GAA-AAC-GCA-TAA-TCTGGG-ACA-GT-3’ and -reverse: 5’-GCG-TGG-TGT-GCT-TAT-TTG-GA-3’. hVEGFR2forward: 5’-AAC-GTG-TCA-CTT-TGT-GCA-AGA-3’ and -reverse: 5’-TTC-CAT-GAGACG-GAC-TCA-GAA-3’. hTBP-forward: 5’-CCA-CTC-ACA-GAC-TCT-CAC-AAC-3’ and -reverse: 5’-CTG-CGG-TAC-AAT-CCC-AGA-ACT-3’

EM of donor eyes

Healthy aged eyes without known ophthalmic diseases were obtained from the Eye Hospital Tuebingen. A glutaraldehyde-fixed tissue sample of the perimacular central region of an AMD donor eye (76 years, male) was obtained from the Foundation fighting Blindness (USA). The AMD eye was investigated by experienced ophthalmologists who stated the AMD type of both eyes including the different types of lesions found in each eye. The pathology

reports also stated the cause of death and further diseases. Written informed consent of all donors for use in medical research and additional approval of the Institutional Review Board of the University of Tuebingen were obtained. The experiments were performed in adherence to the tenets of the Declaration of Helsinki.

The eyes were opened with a circular slit at the limbus and fixed overnight at 4° C in 4% glutaraldehyde in 0.1 M cacodylate buffer (pH 7.4). Then the iris and vitreous were removed and the perimacular region was excised and further prepared for EM as follows. Small pieces (1.5 mm^3) were washed 3 times in 0.1 M cacodylate buffer, postfixed in 1% osmium tetroxide, stained with uranyl acetate and dehydrated in a graded series of ethanol and propylene oxide and embedded in Epon. All EM reagents were purchased from FLUKA (Sigma-Aldrich, St. Louis, MO, USA). Sectioning of the EM blocks was performed using a Reichert Ultracut. Sections were placed on formvar coated Cu slit grids (PLANO (Wetzlar, Germany) and poststained with lead citrate according to standard procedures.

Supplemental tables

Table S-II: RPKM-values for genes expressed by ECs.

gene	isoforms	C-Norm1	C-Norm2	C-Norm3	C-Norm4	Mean
fli1a	ENSDART(	385.612	1076.853	487.3118	502.1537	612.9827
fli1b	ENSDART(	23.76332	84.63377	64.28497	75.73571	62.10444
kdrl	ENSDART(	142.5056	147.8497	243.2102	283.082	204.1619
kdr	ENSDART(	83.91168	49.2961	101.5372	121.7231	89.11701
flt1	ENSDART(	110.7838	28.07704	12.77522	16.95929	42.14883
flt4	ENSDART(	44.13958	26.38287	96.81064	81.4801	62.2033
efnb2a	ENSDART(	39.48331	69.75772	31.60848	37.52966	44.59479
ephb4	ENSDART(	36.65516	47.08962	41.74447	53.90552	44.84869
notch1b	ENSDART(	48.96453	60.56181	51.42755	56.94751	54.47535
dll4	ENSDART(	217.5009	229.5169	178.1177	241.3114	216.6117
nrp1a	ENSDART(	88.07875	70.53185	13.4	20.89838	48.22725
nrp1b	ENSDART(	12.70421	15.09144	5.966144	11.68714	11.36223
pdgfb	ENSDART(	148.643	282.2319	240.7203	257.933	232.382
tek	ENSDART(	51.06712	47.6458	204.3118	242.4483	136.3682
tie1	ENSDART(	7.592462	33.2523	40.72372	40.29371	30.46555

Table S-III: RPKM-values for genes associated with non-vascular retinal cell types

gene	isoforms	C-Norm1	C-Norm2	C-Norm3	C-Norm4	Mean
Pericyte/Smooth muscle cell						
CSPG4	ENSDART0	0.376291	0	0	0	0.094073
desm	ENSDART0	0	0	0	0	0
abcc9	ENSDART0	5.096336	1.603377	6.456484	2.927891	4.021022
kcnj8	ENSDART0	0	0	0	1.660756	0.415189
Neuron						
dbx1b	ENSDART0	0	0	0	0	0
dbx2	ENSDART0	0	0	0	0	0
Astrocyte/Oligodendrocyte						
gfap	ENSDART0	2.377143	0	4.382913	5.721054	3.120277
mbp	ENSDART0	0	0	0	0	0
aldh1a3	ENSDART0	3.801208	0	0	1.688925	1.372533
pax6a	ENSDART0	0	1.93473	0	0	0.483683
pax6b	ENSDART0	0	1.55004	0	0	0.38751
calb2	ENSDART0	0	0	0	0	0

Supplemental figure legends

Figure S-I. Endothelial density in retinal and choroidal vasculatures in zebrafish. A: Western blot using anti-GFP and anti-bactin (control) antibodies on proteins extracted from adult fli1a:EGFP retinas and choroids **B**, **C:** FACS of GFP+ endothelial cells from retinae (**B**) or choroids (**C**). Green dots indicate endothelial cells, blue dots indicate non-endothelial cells. **D:** Quantifications of the cell counts from the experiment shown in **B** and **C**. Results are given as percent endothelial cells compared to the total cell population of the tissue. R: Retina. C: Choroid. n=12-16 fish. **:p<0.01.

Figure S-II. Smooth muscle cell coverage of the choriocapillaris of adult zebrafish. A: Confocal micrographs of adult Kdrl:DsRed;Acta2:EGFP double transgenic zebrafish. Boxed regions are shown in the magnified images below. White arrowheads indicate smooth muscle fiber extensions. Scale-bars indicate 100 μm for retinal vessels and 50 μm for CCs. **B**: Quantification of the percentage of the EGFP+ endothelium that was covered by Acta2+ cells from the experiment shown in **A**. A: Arterial region. C: Capillary region. n=5-10. ***:p<0.001.

Figure S-III. CDH5 and GFAP coverage in the zebrafish. A-B: Confocal micrographs of fli1a:EGFP zebrafish retinal vessels and CCs stained with anti-CDH5 (**A**) or anti-GFAP antibodies. Size-bars indicate 50 μm for retinal vessels and 25 μm for CCs.

Figure S-IV. HIF1α and HIF2α are stabilized in hypoxia. Bright field micrograph of the outer nuclear layer stained with antibodies recognizing HIF1α or HIF2α (positive signals in red) from adult zebrafish treated with normoxia or hypoxia for 10 days.

Figure S-V. High blood pressure is not sufficient to drive PVR. A: Confocal micrographs of CCs from adult vehicle or clenbeuterol treated fli1a:EGFP zebrafish. Size bars indicate 25 μm. **B:** Quantification of number of extravascular columns from experiment shown in **A**. V= vehicle, C= clenbeuterol, NS= non-significant. n= 5-10.

Figure S-VI. Genes containing HREs in the promotor region were not enriched among de-regulated transcripts following 10 days of hypoxia treatment. Lists of Up- or down-regulated genes from hypoxia-exposed zebrafish compared to controls were used to generate lists of orthologous human genes, which in turn were compared to lists of genes from HUVEC cells known to have HIF1a (**A**) or HIF2a (**B**) bound at their promoters following a hypoxic insult[24, 25].

Figure S-VII. Differential cytoskeletal dynamics in choroidal ECs during hypoxia-induced PVR. Functional annotation clusters containing up- and down-regulated genes in endothelial cells from hypoxia-exposed adult zebrafish choroids compared to endothelial cells from normoxia control zebrafish.

Figure S-VIII. Lack of UCHD-associated f-actin bundles in choroidal ECs A: Confocal micrographs of choriocapillaries (CCs) in fli1a:GFF;UAS:RFP;UAS:UCDH-GFP fish in which the fli1a+ endothelium is labeled in red and UCHD+ f-actin bundles in green. **B:** Western blot for GFP- or bactin-expression in choroids of the fish shown in **A**. L: Ladder, C: Choroid.

Figure S-IX. Phalloidin coverage in CCs. Confocal micrographs of adult fli1a:EGFP normoxia- or hypoxia-exposed zebrafish CCs stained with phalloidin. White arrow heads indicates f-actin bundles associated with incomplete/immature transluminal pillars. Size-bars indicate 25 μm.

Figure S-X. Open CCs in hypoxia- or VEGF-treated zebrafish and rats respectively. TEM images showing pathological CCs in hypoxia-treated zebrafish (**A**) or following VEGF-overexpression in rats (**B**). . Boxed regions are shown in magnified images to the right.
F: Fenestrations. ELPs: Endothelial luminal processes.

Figure S-XI. Reduced Collage-fibers and connections between endothelial and vascular mural cells in hypoxia. Transcription electron micrographs of the choriocapillaris in adult zebrafish treated with normoxia or hypoxia. Boxed regions are shown in magnified images below. Yellow arrows point to collagen fibers. White arrows point to connections between endothelial and vascular mural cells. Yellow "Ps" indicate the RPE-side of the extravascular columns, while "Ls" indicate the vascular lumens. T: Tight junction. Size bar indicate 5 μm.

Figure S-XII. VEGFR-signaling in the CCs is activated by hypoxia. A: Fluorescent micrographs of cross sections through the retinal vessels (RVs) or choriocapillaris (CCs) stained with antibodies recognizing phosphorylated VEGFR2 (positive signals in white) and counterstained with DAPI (nuclei shown in blue) from adult zebrafish treated with normoxia or hypoxia for 10 days. **B**: Relative expression of known VEGF-target genes in hypoxia-treated fish compared to normoxia treated fish extracted from the RNA-sequencing data in Table S4

Supplemental Figure I

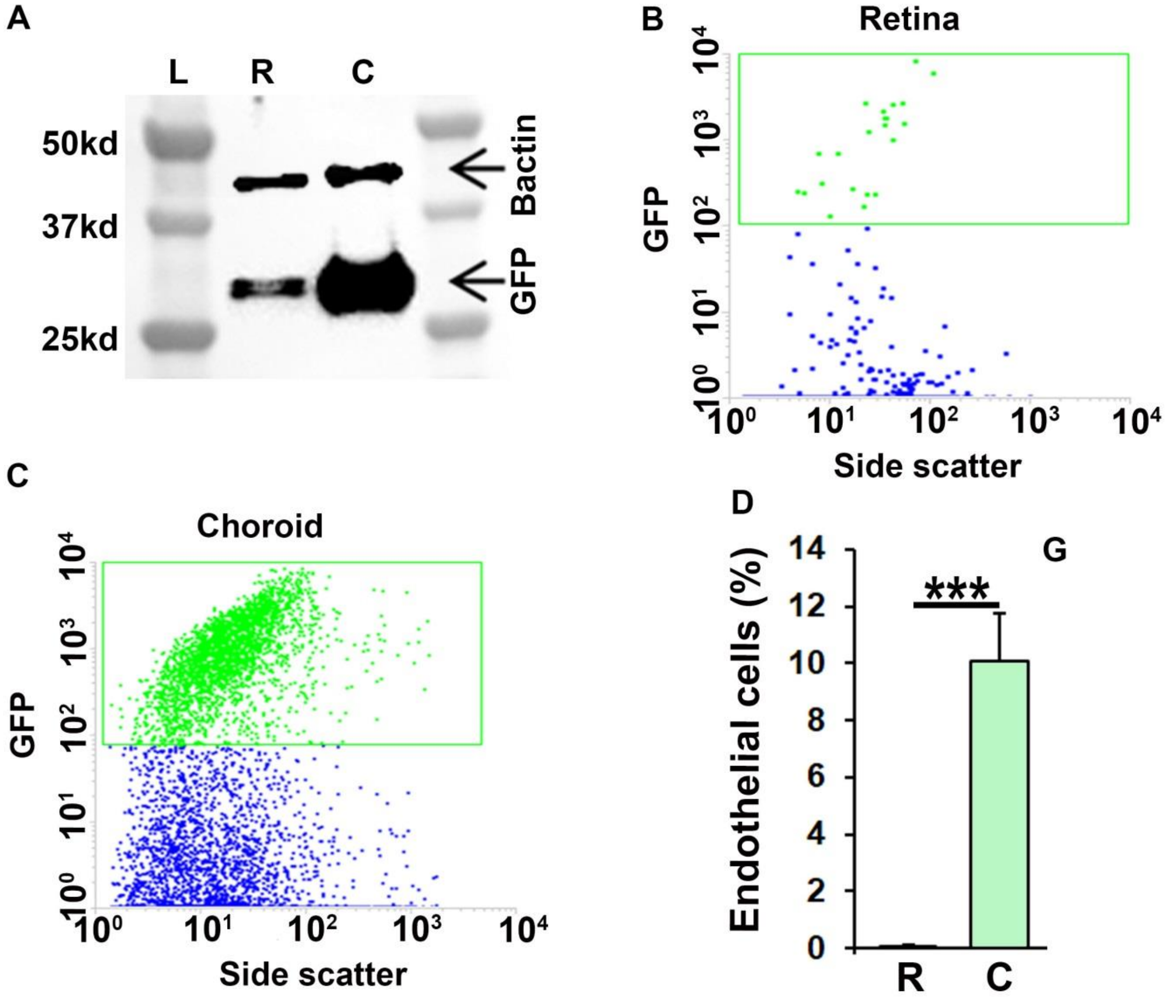

Supplemental Figure II

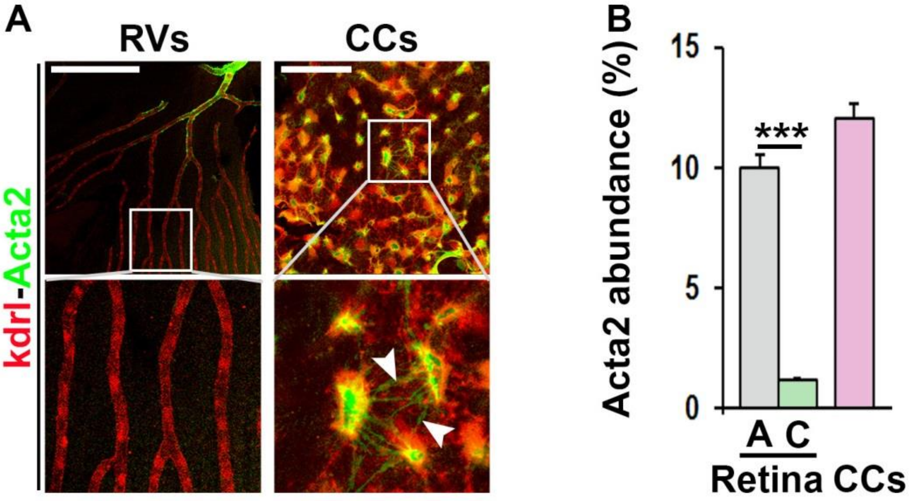

Supplemental Figure III

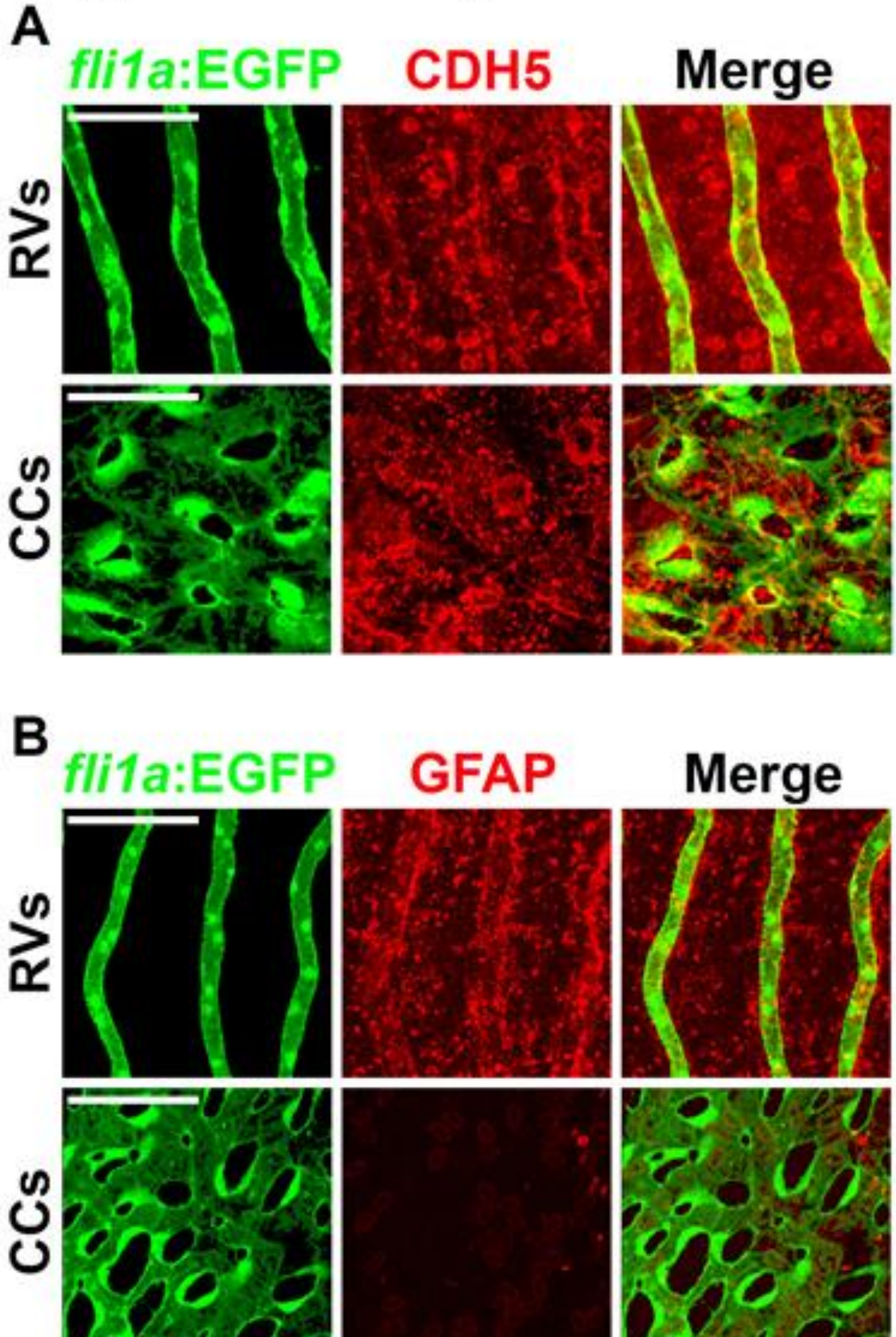

Supplemental Figure IV

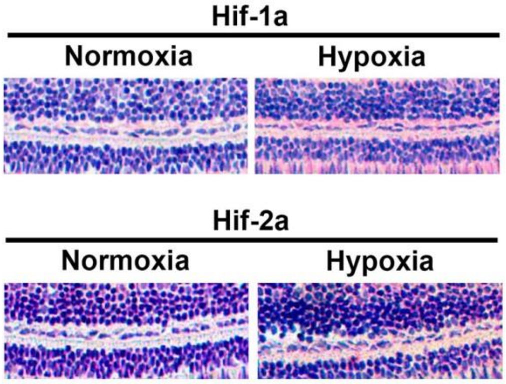

Supplemental Figure V

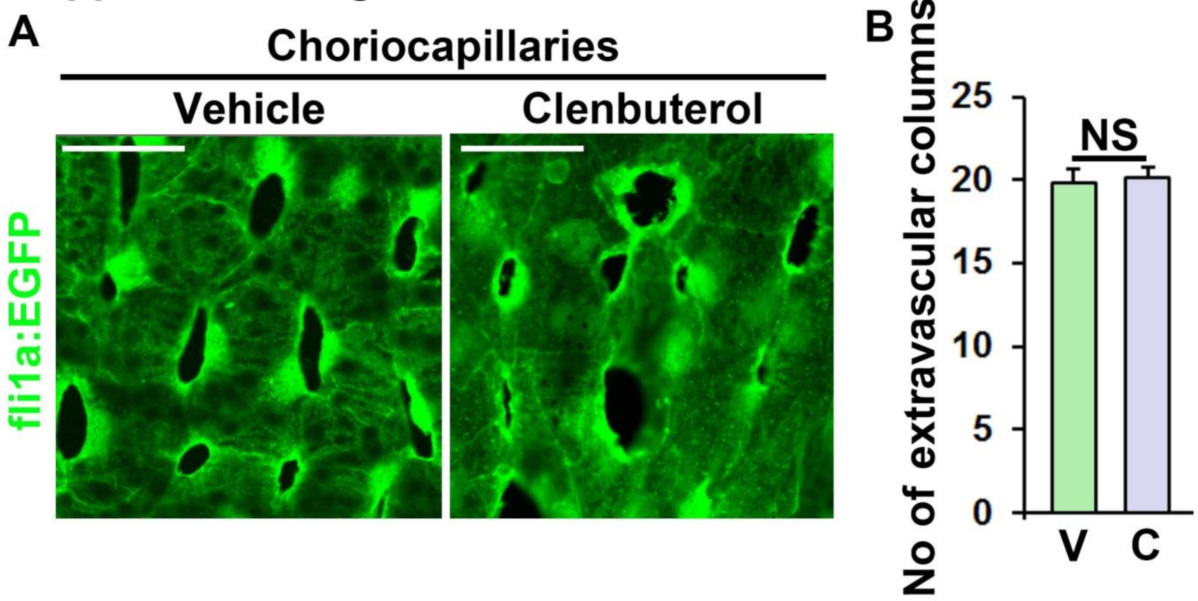

Supplemental Figure VI

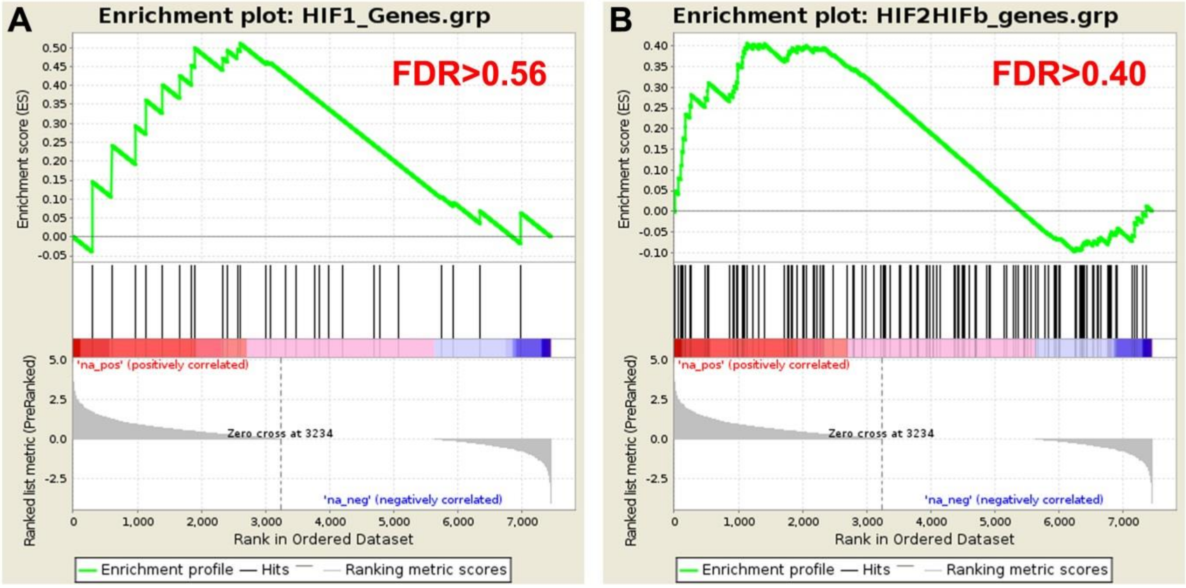

Supplemental Figure VII

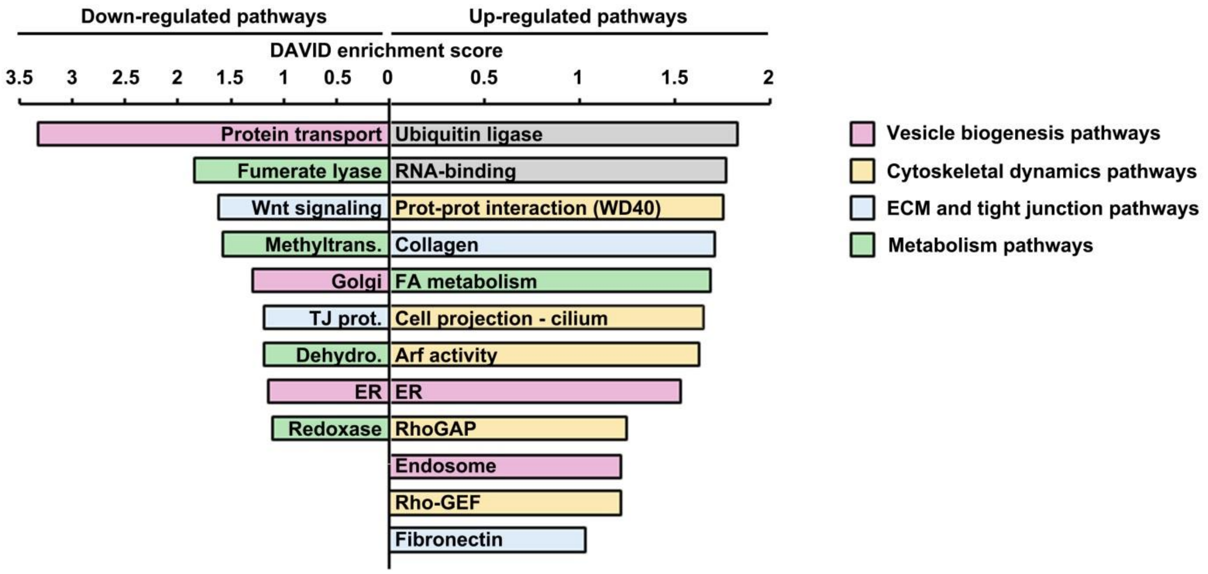

Supplemental Figure VIII

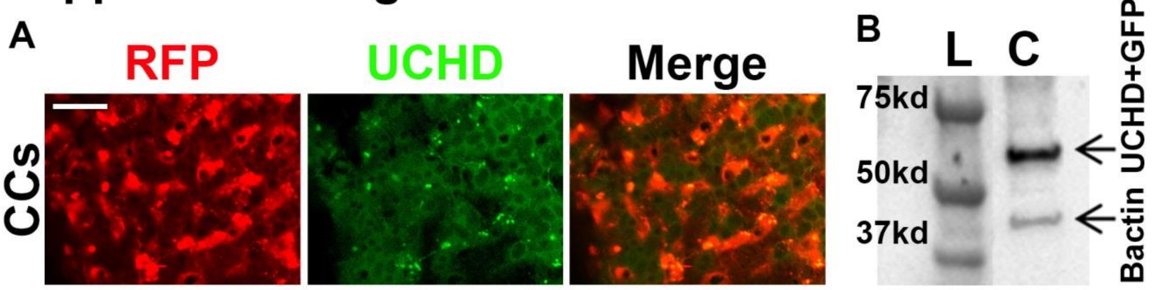

Supplemental Figure IX

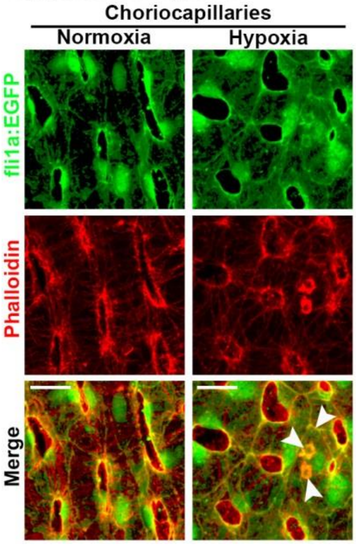

Supplemental Figure X

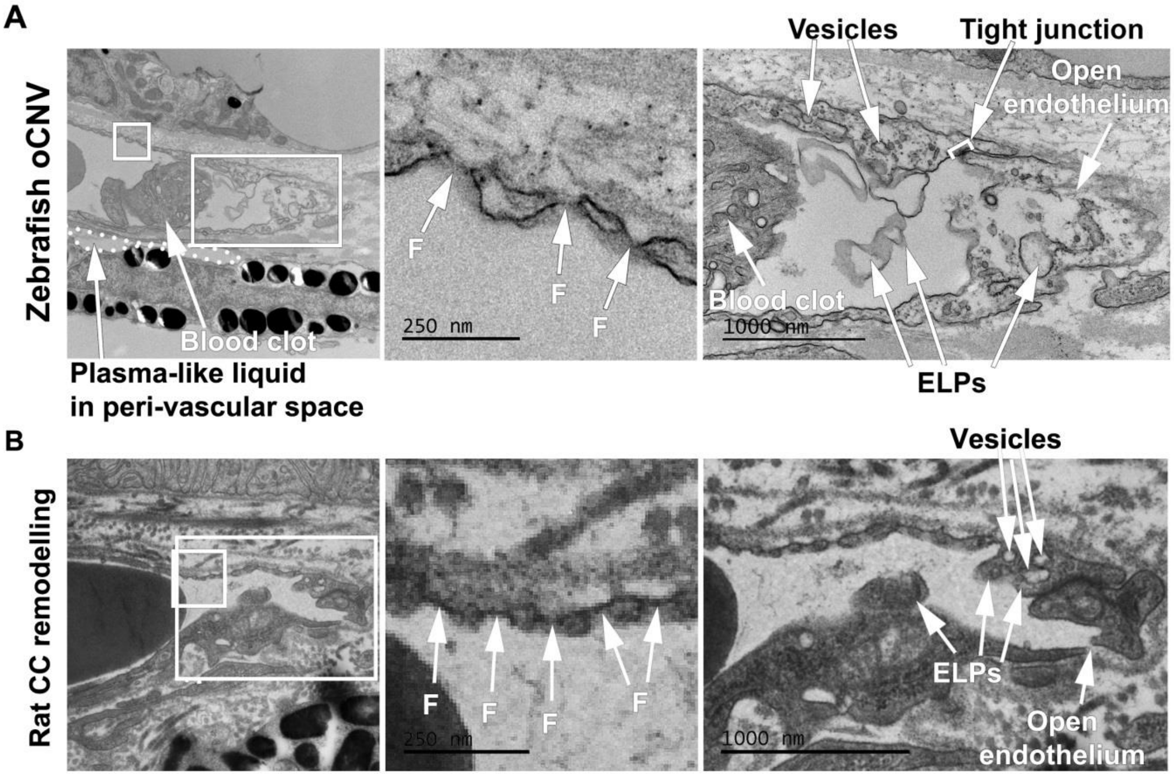

Supplemental Figure XI

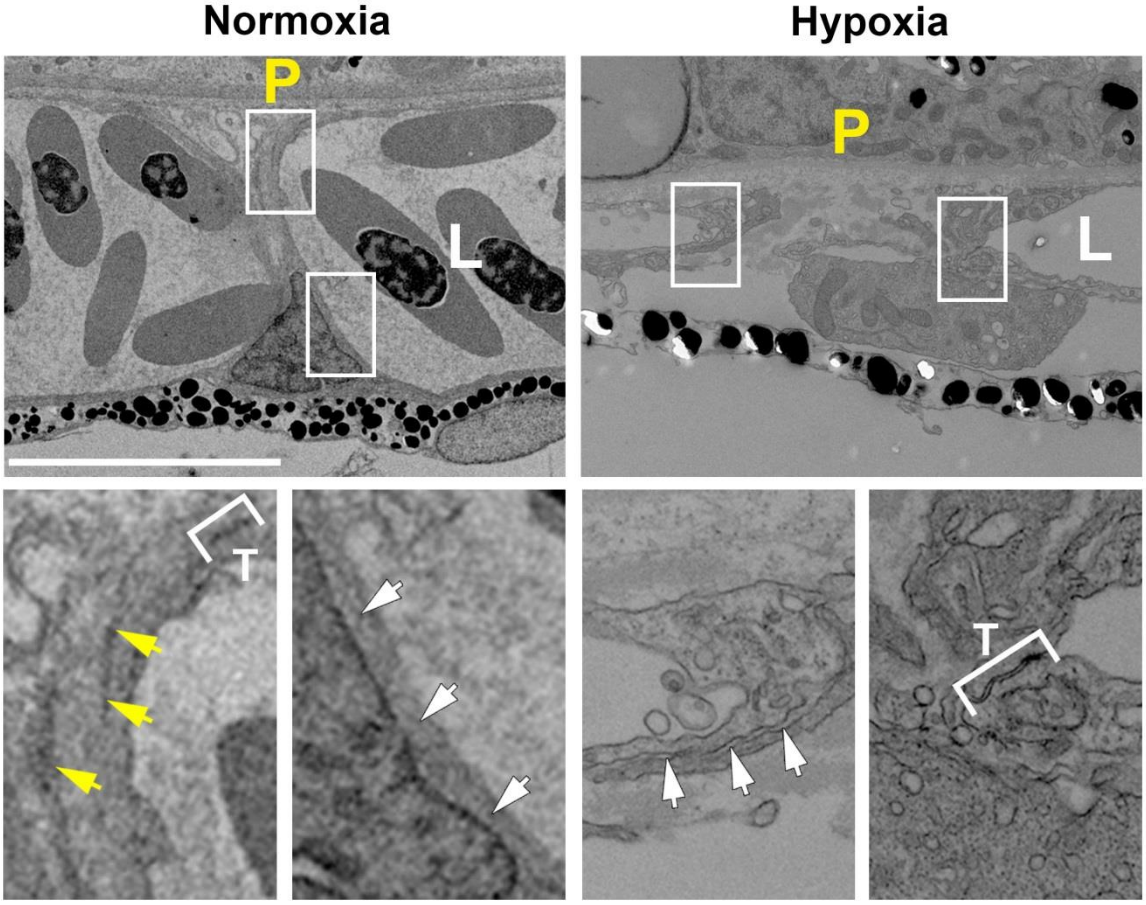

Supplemental Figure XII

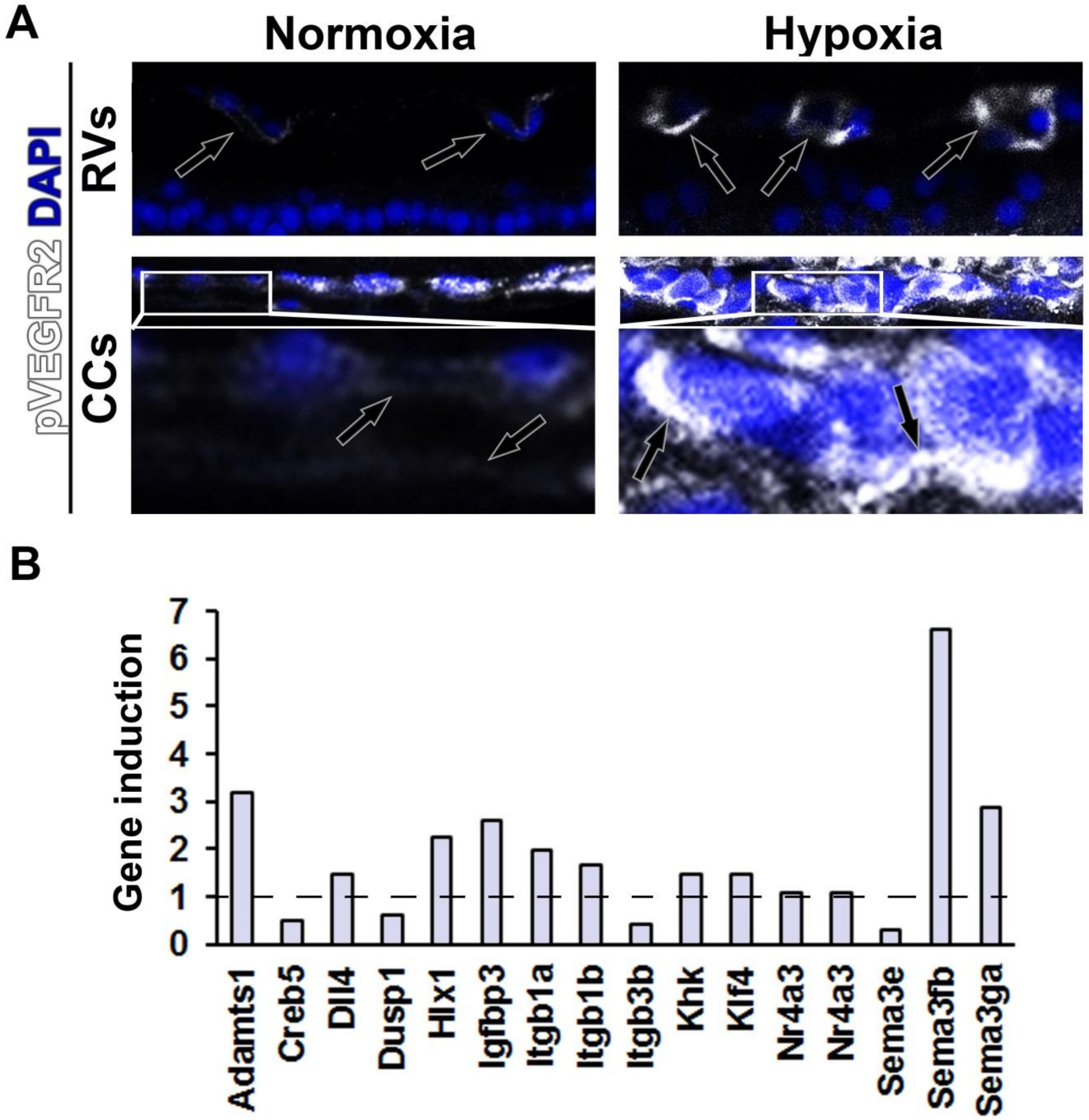

Supplement: Supplementary file 1 [file atv-39-1402-s001.pdf]
