# Supplementary material for: Intussusceptive Vascular Remodeling Precedes Pathological Neovascularization
Source: Arterioscler Thromb Vasc Biol. 2019 May 9;39(7):1402–18. doi: 10.1161/ATVBAHA.118.312190 (PMC6636809; doi:10.1161/ATVBAHA.118.312190)
Supplement: Supplementary file 2 [file atv-39-1402-s002.pdf]

Supplemental Table S-I: Major resource table

Zebrafish strains used in this study

Strain name	Affected promoter/protein	Cell type/structure	Fluorophore	Ref.
Tg(fli1a:EGFP)^{y1}	*fli1a*	Endothelial	Green	54
Tg(kdrl:EGFP)^{s843}	*kdrl*	Endothelial	Green	55
Tg(kdrl:DsRed2)^{pd27}	*kdrl*	Endothelial	Red	56
Tg(acta2:EGFP)^{ca7}	*acta2*	Smooth muscle	Green	57
Tg(tagln:EGFP)^{p151}	*tagln*	Smooth muscle	Green	58
Tg(gata1a:DsRed2)	*gata1a*	Erythroblast	Red	59
Tg(fli1ep:Gal4FF)^{ubs3}	*fli1a*	Endothelial	No colour	60
Tg(UAS:RFP)	UAS	All (ubiquitous)	Red	61
Tg(UAS:VE-Cadherin-EGFP)^{ubs17}	VE-Cadherin (*cdh5*)	Tight junctions	Green	61
Tg(UAS:EGFP-ZO.1)^{ubs5}	ZO.1	Tight junctions	Green	62
Tg(UAS:EGFP-ECHD)^{ubs19}	fActin	Cytoskeleton	Green	63
Tg(pdgfrb:mCitrine)	*pdgfrb*	Pericytes	Green	64
hif1aa$^{-/-}$;hif1ab$^{-/-}$	*hif1aa, hif1ab*	All (ubiquitous)	Null-mut.	65
hsp70:VEGFAa-DN	VEGF-Aa	All (ubiquitous)	Dominant negative isoform	66

Primers used in this study

**Primer name**	**Sequence (5' -> 3')**
hVegfa-forward	AGGGCAGAATCATCACGAAGT
hVegfa-reverse	AGGGTCTCGATTGGATGGCA
hPlgf-forward	GAACGGCTCGTCAGAGGTG
hPlgf-reverse	ACAGTGCAGATTCTCATCGCC
hVegfr1-forward	GAAAACGCATAATCTGGGACAGT
hVegfr1-reverse	GCGTGGTGTGCTTATTTGGA
hVegfr2-forward	AACGTGTCACTTTGTGCAAGA
hVegfr2-reverse	TTCCATGAGACGGACTCAGAA
hTbp-forward	CCACTCACAGACTCTCACAAC
hTbp-reverse	CTGCGGTACAATCCCAGAACT

Antibodies used in this study

**Target antigen**	**Brand**	**Catalog #**	**Dilution**
ZO-1	Invitrogen	61-7300	1:250
GFAP	Sigma	SAB2702474	1:50
VE-Cadherin	Ref: 49	Ref: 49	1:200
Col-I	Abcam	GR3211489	1:200

HIF1a	Novus	NB100-134	1:600
HIF2a	Novus	NB100-122	1:300
pVEGFR2	Abcam	Ab5473	1:50
βActin	Invitrogen	PA1-21167	1:2000
GFP	Invitrogen	A-11122	1:2000
PECAM/CD31	R&D Biosciences	AF3628	1:200
VEGFR1	Imclone	MF1	1:200
VEGFR2	Imclone	DC101	1:200
Goat-anti-rabbit Alexa-555	Life technologies	A21428	1:200
Goat-anti-rabbit Alexa-647	Invitrogen	A21345	1:2000
Donkey-anti-rabbit Alexa-555	Invitrogen	A31572	1:400
Donkey-anti-goat Alexa-647	Invitrogen	A21447	1:200
Goat-anti-rat Alexa-555	Invitrogen	A21434	1:400
