# Supplementary figures and images for: Intussusceptive Vascular Remodeling Precedes Pathological Neovascularization
Source: Arterioscler Thromb Vasc Biol. 2019 May 9;39(7):1402–18. doi: 10.1161/ATVBAHA.118.312190 (PMC6636809; doi:10.1161/ATVBAHA.118.312190)

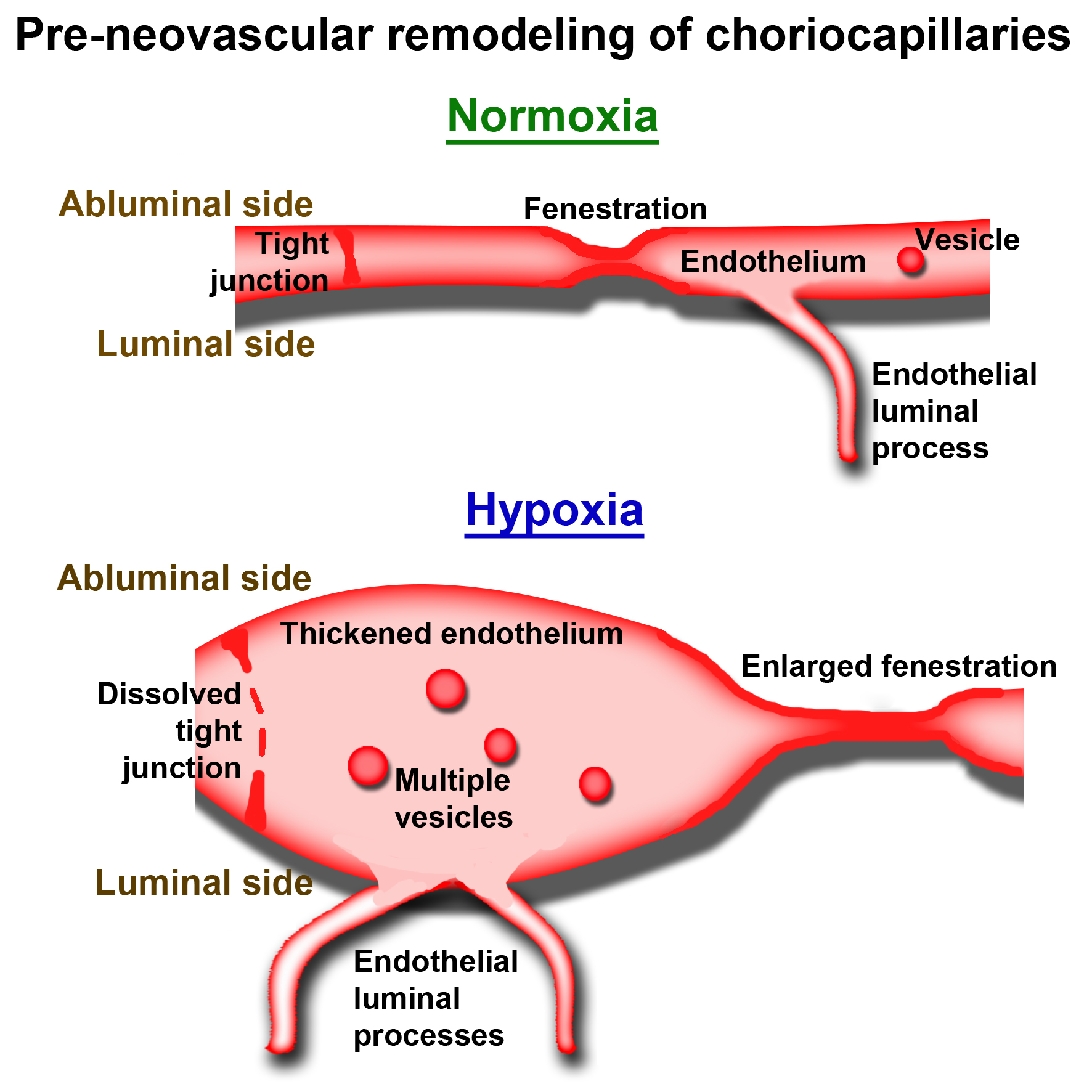

Supplement: Supplementary file 3 [file atv-39-1402-s003.jpg]
